# Polystyrene nanoplastics-induced lung epithelial cells ferroptosis promotes pulmonary fibrosis via YY1/FTL axis

**DOI:** 10.1016/j.mtbio.2025.102738

**Published:** 2025-12-24

**Authors:** Wenxia Bu, Yueyuan Jin, Yifan Zhou, Fengxu Wang, Dongnan Zheng, Rongzhu Liu, Xuehai Wang, Mengjiao Yu, Shan Bao, Rui Zhao, Jinlong Li, Xiaoyu Zhou, Jian Feng, Xinyuan Zhao, Demin Cheng

**Affiliations:** a Department of Respiratory and Critical Care Medicine, Affiliated Hospital of Nantong University, School of Public Health, Nantong University, Nantong, 226001, China; b China CDC Key Laboratory of Environment and Population Health, National Institute of Environmental Health, Chinese Center for Disease Control and Prevention, Beijing, 100021, China; c Department of Pulmonary and Critical Care Medicine, The First Affiliated Hospital of Soochow University, Suzhou, 215006, China; d Department of Respiratory, Wuxi Eighth People’s Hospital, Wuxi, 214000, China; e School of Public Health, North China University of Science and Technology, Tangshan, Hebei, 06321, China

**Keywords:** Polystyrene nanoplastics, Ferroptosis, Pulmonary fibrosis, YY1, FTL

## Abstract

Nanoplastics (NPs) have emerged as pervasive environmental pollutants, and polystyrene nanoplastics (PS-NPs) have been increasingly implicated in pulmonary toxicity and fibrosis. Here, we established both an in vitro BEAS-2B bronchial epithelial injury model and an in vivo mouse model by repeated oropharyngeal aspiration of PS-NPs. We observed pronounced pulmonary fibrosis and ferroptotic death of alveolar type II cells. Integrated transcriptomic and proteomic profiling highlighted enrichment of the ferroptosis pathway, and in vitro and in vivo experiments confirmed that PS-NPs suppress GPX4 while inducing FTL, thereby driving lipid peroxidation. Mechanistically, YY1 binds the FTL promoter to repress its expression, and YY1 knockdown alleviates ferroptosis and fibrosis. Our findings identify YY1/FTL-mediated ferroptosis as a key mechanism by which PS-NPs induce pulmonary fibrosis.

## Graphical abstract

**Figure:**
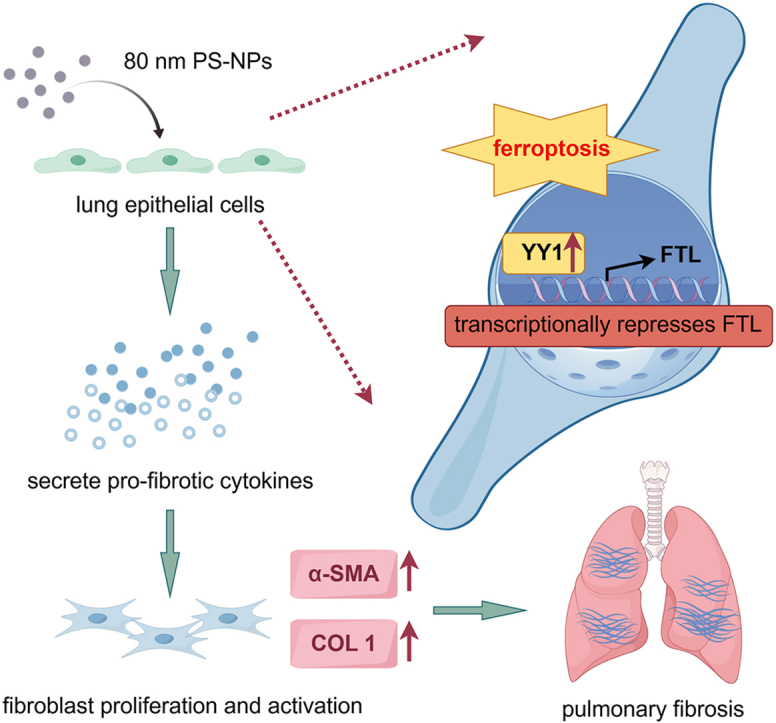

## 1 Introduction

Plastic pollution has emerged as a critical global environmental challenge, with microplastics (MPs) and nanoplastics (NPs) ubiquitous environmental contaminants [1]. These particles, particularly NPs, are of increasing concern because they can cross biological barriers and accumulate in human tissues [2]. Both experimental and epidemiological studies have associated exposure to NPs with a range of adverse health outcomes, including respiratory, gastrointestinal, and neurological disorders [3,4]. Despite growing awareness, the molecular mechanisms underlying these health impacts remain poorly defined, underscoring the need for further mechanistic research to clarify their biological effects and to inform strategies for mitigating the health risks associated with plastic pollution.

Polystyrene nanoparticles (PS-NPs) are among the most widespread nanoplastic contaminants in natural environments [5]. Polystyrene is extensively used in diverse consumer products, including household goods, toys, thermal paper, and medical supplies [6,7], leading to the ubiquitous release of PS-NPs into the environment. Recent studies have detected PS-NPs in human lung tissues, raising concern about their potential contribution to respiratory disease pathogenesis [8,9]. Experimental and observational evidence indicate that inhalation of airborne PS-NPs is associated with lung injury and other respiratory complications [10]. As PS-NPs are more easily inhaled and can penetrate deeper into the distal airways and alveolar regions of the respiratory system compared to larger particles [11], their impact on human health, particularly the lungs, warrants urgent and systematic investigation and further research.

Ferroptosis is a regulated form of cell death driven by iron-dependent lipid peroxidation, which plays a significant role in maintaining cellular homeostasis and is implicated in a wide range of diseases [12,13]. This process is characterized by the excessive accumulation of lipid peroxides due to the depletion of glutathione (GSH) and reduced activity of the lipid repair enzyme glutathione peroxidase 4 (GPX4), leading to oxidative damage and cell death [14,15]. This process is tightly governed by iron metabolism, particularly the ferritin light chain (FTL) and ferritin heavy chain 1 (FTH1), which are the crucial proteins responsible for iron storage [[16], [17], [18]]. Dysregulation of FTL can result in intracellular iron overload, which in turn amplifies oxidative stress and lipid peroxidation, ultimately initiating ferroptosis [19]. Recent studies highlight the involvement of ferroptosis in lung diseases and suggest that ferroptosis may also contribute to lung injury caused by environmental pollutants, including nanoplastics [20,21]. Consistent with these observations, emerging evidence suggests a connection between PS-NPs and ferroptosis-mediated lung injury and fibrosis [22], highlighting the need for further investigation into the mechanisms by which PS-NPs contribute to disease progression.

In the current study, we demonstrate that PS-NPs trigger an early, compensatory upregulation of FTL in lung epithelial cells, which is transcriptionally regulated by YY1 through its binding to the FTL promoter, thereby promoting ferroptosis and pulmonary fibrosis in vitro and in vivo. Furthermore, genetic knockdown of YY1 significantly attenuated ferroptosis while further enhancing FTL expression in lung epithelial cells. Our in vivo data also indicated that delivery of an adenovirus-mediated Yy1 shRNA vector under the control of the surfactant protein C promoter protected mice from PS-NPs-induced pulmonary fibrosis. Together, our data provide novel mechanistic insight into PS-NPs-induced ferroptosis in lung epithelial cells and elucidate a key pathway underlying the respiratory toxicity of PS-NPs.

## 2 Materials and methods

### 2.1 Characterization of PS-NPs

The polystyrene nanoplastics (PS-NPs, 80 nm) were obtained from the Base Line Chrom Tech Research Centre (Tianjin, China). Before administration, the PS-NPs were subjected to ultrasonication for 30 min to ensure a homogeneous suspension. The morphologies of PS-NPs were observed by scanning electron microscopy (SEM, ZEISS, GeminiSEM 300, Germany) and transmission electron microscopy (TEM, FEI Talos 200X, America).

### 2.2 Cell culture and treatment

The human bronchial epithelial cell (BEAS-2B) and human embryonic lung fibroblast (MRC-5) were commercially purchased from the American Type Culture Collection (ATCC, Manassas, VA, USA). BEAS-2B and MRC-5 cells were cultured in Dulbecco’s modified Eagle’s medium (DMEM, Life Technologies/Gibco, Grand Island, NY, USA). All of the culture media contained 10 % fetal bovine serum (FBS, Gibco, 1027-106) and antibiotics (penicillin and streptomycin, Life Technologies/Gibco, Gaithersburg, MD). All cells were cultured at 37 °C in a 5 % CO2 atmosphere.

For in vitro experiments, BEAS-2B cells were exposed to PS-NPs (200 μg/mL) for 24 h unless otherwise specified. To assess the contribution of different cell death pathways, cells were assigned to the following treatment groups: control, PS-NPs alone, PS-NPs + Ferrostatin-1 (Fer-1), PS-NPs + Necrostatin-1, and PS-NPs + Z-VAD-FMK. Fer-1 (SML0583, 10 μM), Necrostatin-1 (30 μM), and the pan-caspase inhibitor Z-VAD-FMK (20 μM) were applied at concentrations previously reported to be effective in BEAS-2B cells [23,24]. Cell viability in each group was determined using the CCK-8 assay to evaluate the extent of rescue from PS-NPs-induced cytotoxicity.

In rescue experiments targeting ferroptosis and YY1, YY1 siRNA (5 μM) was transfected into BEAS-2B cells 24 h before PS-NPs exposure, and Fer-1 (10 μM) was added 2 h before PS-NPs treatment. For YY1 knockdown experiments, siNC (negative control) denotes cells transfected with a non-targeting control siRNA. To investigate the role of FTL, BEAS-2B cells were transfected with siRNA targeting FTL (siFTL) or siNC and subsequently treated with PS-NPs, generating siNC + PS-NPs and siFTL + PS-NPs groups for comparison. The sequences of all siRNAs are provided in Supplementary Table 1.

For the preparation of BEAS-2B cell–derived conditioned media, BEAS-2B cells were subjected to the treatments described above (PS-NPs, YY1 siRNA, Fer-1, and the corresponding controls). After treatment, cells were washed twice with PBS and further cultured in FBS-free DMEM for 24 h. The supernatant was then collected and used to culture MRC-5 cells for 24 h.

For mRNA stability assays, BEAS-2B cells were treated with vehicle (control) or PS-NPs for 24 h, followed by the addition of the transcription inhibitor actinomycin D (ActD). Cells were harvested at 0, 9, 12, and 24 h after ActD treatment, and total RNA was extracted for quantitative RT–PCR analysis of FTL mRNA. FTL mRNA levels at each time point were normalized to the 0 h value and to the internal reference gene to evaluate PS-NPs-induced changes in FTL mRNA decay.

For protein stability assays using cycloheximide (CHX), two independent experimental series were performed. In the first series, BEAS-2B cells were assigned to control and PS-NPs treatment groups, followed by addition of CHX to block de novo protein synthesis. Cells were collected at 0, 3, 6, and 9 h after CHX treatment, and total protein was extracted for Western blot analysis of FTL. In the second series, BEAS-2B cells were transfected with siNC or siYY1 and subsequently exposed to PS-NPs to generate siNC + PS-NPs and siYY1+PS-NPs groups. After PS-NPs treatment, CHX was added (0 h), and cells were harvested at 0, 3, 6, and 9 h for Western blot detection of FTL. The time-dependent decrease in FTL protein abundance in each series was quantified to assess FTL protein degradation under the respective conditions.

### 2.3 Animal study design

The mouse model of pulmonary fibrosis was established as previously reported by our group [25]. Male C57BL/6 mice (6–8 weeks) were obtained from the China National Laboratory Animal Resource Center (Shanghai, China) and housed under standard conditions (22 ± 2 °C, 40–50 % humidity, 12 h light/dark cycle) with free access to food and water. Based on previously reported upper-bound estimates of airborne nanoplastic inhalation in humans (6.67 × 10^16^ particles day^−1^) and the corresponding rat-equivalent dose derived by lung surface area scaling (4.43 × 10^14^ particles day^−1^) [26,27], we further extrapolated the human exposure to mice using the same lung surface area–based dosimetry approach (62.7 m^2^ for humans vs. 0.05 m^2^ for mice), yielding a theoretical mouse upper-bound dose of approximately 5.3 × 10^13^ particles day^−1^. To avoid unrealistically high lung burdens while maintaining a high-end, environmentally relevant exposure, we selected a slightly lower but same-order-of-magnitude dose of 1.88 × 10^13^ PS-NPs particles day^−1^, which corresponds to the working concentration of 25 μg/μL used in our 4-week subacute exposure protocol. Mice were randomly allocated into two groups (n = 8 per group): a control group receiving sterile PBS and an exposure group administered 25 μg/μL PS-NPs. Before each administration, the PS-NPs suspension was prepared in sterile PBS, ultrasonicated for 15 min, and warmed to 37 °C. Under anesthesia with 2 % isoflurane in an upright position, mice received oropharyngeal aspiration three times per week, with 50 μL of the nanoparticle suspension deposited onto the posterior tongue. Body weight and general health status were monitored throughout the experiment. For the Yy1 shRNA adenovirus intervention group (n = 8), mice were administered Yy1 shRNA adenovirus via oropharyngeal aspiration. The NC (negative control) group (n = 8) received a control shRNA adenovirus using the same protocol. Four weeks later, mice were treated with PS-NPs. At the experimental endpoint, the mice were sacrificed under anesthesia. Lung tissues were obtained and stored at −80 °C for further analysis. All animal studies were approved by the Guiding Principles for the Use of Animals at Nantong University.

### 2.4 Histopathology

Lung tissues from the right lung were fixed in 4 % paraformaldehyde (PFA), embedded in paraffin, cut into 4 μm sections, and then put on slides. Lung sections were stained with H&E staining according to the manufacturer’s instructions to assess histopathological changes. Additionally, Masson and Sirius red staining were employed to evaluate the fibrotic areas within lung sections.

### 2.5 Immunohistochemistry (IHC)

Paraffin-embedded lung sections were deparaffinized, rehydrated through graded ethanol, and subjected to heat-induced antigen retrieval. After blocking with 5 % goat serum, sections were incubated overnight at 4 °C with the following primary antibodies: α-SMA (1 : 200), GPX4 (1 : 200), FTL (1 : 200), FTH1 (1 : 200), and YY1 (1 : 200). After washing, slides were exposed to the appropriate HRP-conjugated secondary antibody and developed with 3,3′-diaminobenzidine (DAB; Sigma) until the desired intensity was reached. Color development was terminated in running tap water, nuclei were counterstained with hematoxylin, and sections were dehydrated, cleared, and coverslipped for microscopic evaluation. Quantitative analysis of DAB-positive staining (including positive area and mean optical density) was performed using ImageJ software (NIH).

### 2.6 Western blot and antibodies

Total protein from lung tissues and cells was isolated using RIPA lysis buffer (Beyotime, #P1046, China) supplemented with protease inhibitors. Protein concentrations were determined using the bicinchoninic acid (BCA) assay (Beyotime, #P0009, China). For each sample, approximately 30 μg of protein was separated by electrophoresis on either a 10 % or 15 % SDS–PAGE gel. Following electrophoresis, the proteins were transferred onto a polyvinylidene difluoride (PVDF, Millipore Corporation, Billerica, #MA 01821, USA) membrane and then blocked at room temperature with 5 % skim milk. The membrane was incubated with rabbit anti-GPX4 (1:1000; Abways, #CY6959), rabbit anti-FTH1 (1:1000; Abways, #CY5648), rabbit anti-FTL (1:1000; Proteintech, #10727-1-AP), anti-α-SMA (1:1000; Abways, #CY5295), anti-COL1 (1:1000; Proteintech, #14695-1-AP), anti-YY1 (1:1000; Cell Signaling, #46395), and rabbit anti-β-actin (1:1000; Sigma, #sc-69879) antibodies and incubated at 4 °C overnight. Then, the membranes were incubated with a secondary antibody (goat anti-rabbit IgG from Sigma, #A0545, USA, 1:20000) at room temperature for 1 h. Finally, the protein bands were visualized and captured using the Tanon-5200 imaging system (Tanon Company, Shanghai, China). The intensity of the bands was quantified using ImageJ software (National Institutes of Health, Bethesda, MD, USA), with β-actin serving as the reference for normalization.

### 2.7 Real-time quantitative PCR (qRT-PCR) analysis

Total RNA was isolated from lung tissues using TRIzol reagent (Takara, Japan). The RNA concentration was measured using a NanoDrop 8000 spectrophotometer (Thermo Fisher Scientific, Wilmington, DE, USA) by assessing absorbance at 260 nm and 280 nm. cDNA synthesis was performed with an Omniscript reverse transcription kit (Takara, Japan) and Oligo dT primer, following the manufacturer’s protocol. Gene expression levels were analyzed by RT-qPCR using TB green (Takara, Japan) on Roche LightCycler 480 system. Primer sequences are listed in Supplementary Table 2. The relative mRNA expression was quantified using the 2–ΔΔCt method, with GAPDH mRNA serving as the internal reference for normalization.

### 2.8 Transcriptomic and proteomic analysis

BEAS-2B cells were cultured in high-glucose DMEM with 10 % FBS and 1 % penicillin–streptomycin at 37 °C/5 % CO_2_, and upon reaching 70–80 % confluence were either maintained in complete medium (control) or treated with 200 μg/mL polystyrene nanoparticles for 24 h; afterward, cells were washed twice with ice-cold PBS and processed for RNA-seq and proteomics. For transcriptomics, total RNA was isolated by TRIzol™, quality-checked (A_260_/_280_ 1.8–2.0, RIN ≥7), and libraries were prepared with NEBNext® Ultra™ II before 150 bp paired-end sequencing on NovaSeq 6000. Reads were filtered (FastQC, Trimmomatic), aligned to hg38 via STAR, counted by featureCounts, and differential expression assessed by DESeq2 (|log_2_FC| ≥ 1, FDR <0.05). For proteomics, cell lysates in RIPA buffer were quantified (BCA), integrity verified by SDS–PAGE, reduced (DTT), alkylated (IAA), and digested with trypsin; peptides were desalted on C18 columns and analyzed by LC–MS/MS on an Orbitrap Exploris 480 (MS1 60,000; MS2 15,000; exclusion 30 s). Data were processed with MaxQuant against the UniProt human database (FDR ≤1 %) for LFQ quantification, and statistical/enrichment analyses were performed in Perseus and ClusterProfiler (|log_2_FC| ≥ 1, P < 0.05).

### 2.9 Measurement of hydroxyproline

The hydroxyproline content in lung tissues and serum was measured using a hydroxyproline assay kit (Jiancheng Bioengineering Institute, Nanjing, China, A030-2-1). Lung tissue samples were hydrolyzed and centrifuged once the pH was adjusted to a range of 6.0–6.8. Following this, the hydroxyproline levels were determined by absorbance measurement at 560 nm, as per the manufacturer’s guidelines. The results were expressed as micrograms of hydroxyproline per gram of wet lung tissue weight.

### 2.10 Immunofluorescent

Paraffin lung sections (or fixed cell coverslips) were processed with a dual-marker TSA kit (AFIHC023, Aifang Bio, China). Sections were de-waxed, rehydrated, and subjected to citrate-buffer antigen retrieval, then quenched for endogenous peroxidase and blocked with 5 % goat serum. Cycle 1: slides were incubated overnight at 4 °C with rabbit anti-SP-C (1 : 500, Affinity DF6647), followed by HRP-polymer secondary antibody and development with red TSA fluorophore. After heat-mediated antibody stripping, peroxidase blocking, and serum re-blocking, Cycle 2 was performed with primary antibodies against GPX4, FTL, or YY1 (each 1 : 200), developed with green TSA fluorophore. Nuclei were counterstained with DAPI, and slides were dehydrated, cleared, and mounted for confocal imaging (Nikon, Japan). Fluorescence intensity and co-localization of the dual markers were quantitatively analyzed using ImageJ software (NIH).

### 2.11 Biochemical evaluation

To assess enzyme activity, lung tissue samples were homogenized and centrifuged at 3500 rpm for 15 min. The homogenate was collected and utilized for evaluating ferroptosis-related markers, such as superoxide dismutase (SOD, A001-3), malondialdehyde (MDA, A003-2), and glutathione peroxidase (GSH, A006-2). The levels of SOD and GSH activity, as well as MDA concentration, were determined using commercially available assay kits from the Nanjing Jiancheng Institute of Biological Engineering (Nanjing, China), following the provided protocols. All steps were carried out at 4 °C to ensure sample stability.

### 2.12 Lipid peroxidation assessed by C11-BODIPY581/591

BEAS-2B cells were seeded in 6-well plates and cultured with the respective treatments. Then, the cells were incubated with 10 μM C11-BODIPY (RM02821, ABclonal) for 30 min at 37 °C, followed by three washes with PBS. The red and green fluorescence signals within the cells were captured using a fluorescence microscope.

### 2.13 Iron detection

The iron levels in lung tissues were measured using an Iron Colorimetric Assay Kit (Elabscience, E-BC-K139-M), following the provided protocol. The absorbance was measured at 520 nm with a microplate reader. To evaluate intracellular iron, a FerroOrange fluorescent probe (MX4559-48UG, Serve Life Science) was employed to detect Fe2+. Cells were washed and treated with 1 μM FerroOrange for 1 h, then examined under a fluorescence microscope.

### 2.14 Chromatin immunoprecipitation (ChIP)

ChIP assays were performed using the Smart-ChIP ChIP Kit (Engibody, cat. no. ChIP-1001) according to the manufacturer’s instructions. Briefly, cells were crosslinked in 1 % formaldehyde at room temperature for 10 min, and the reaction was quenched by addition of the supplied 10 × stop solution. After washing with ice-cold PBS, cells were collected and lysed in Chromatin Extract Buffer, then incubated on ice to release nuclei. Nuclear lysates were sonicated to shear chromatin to an average size of approximately 200–500 bp, and debris was removed by centrifugation. An aliquot of chromatin (1 % of the total) was saved as input, and the remaining supernatant was incubated overnight at 4 °C with ChIP grade primary antibody (anti-YY1) or the normal IgG control, followed by capture with Protein A/G magnetic beads. Immunocomplexes were washed sequentially with ChIP Wash Buffers 1–4, and bound chromatin was eluted with ChIP Elution Buffer supplemented as recommended by the manufacturer. Crosslinks were reversed by incubation at elevated temperature in the presence of Proteinase K, followed by RNase A treatment and DNA purification using the kit DNA precipitation reagent and ethanol. The recovered DNA was resuspended in nuclease free water and analyzed by ChIP qPCR using the 2 × real time PCR master mix included in the kit and primers targeting the indicated promoter regions. Enrichment was calculated relative to input and isotype IgG controls.

### 2.15 Statistical analysis

Data analysis was conducted using GraphPad Prism 6.01 (GraphPad Software Inc., San Diego, CA, USA). For comparisons involving two groups, Student’s t-test was applied, while one-way ANOVA with Tukey’s post hoc test was used for analyses involving three or more groups. Results are reported as mean ± SD, and statistical significance was defined as a p < 0.05.

## 3 Results

### 3.1 Exposure to PS-NPs caused pulmonary fibrosis

**Fig. 1:**
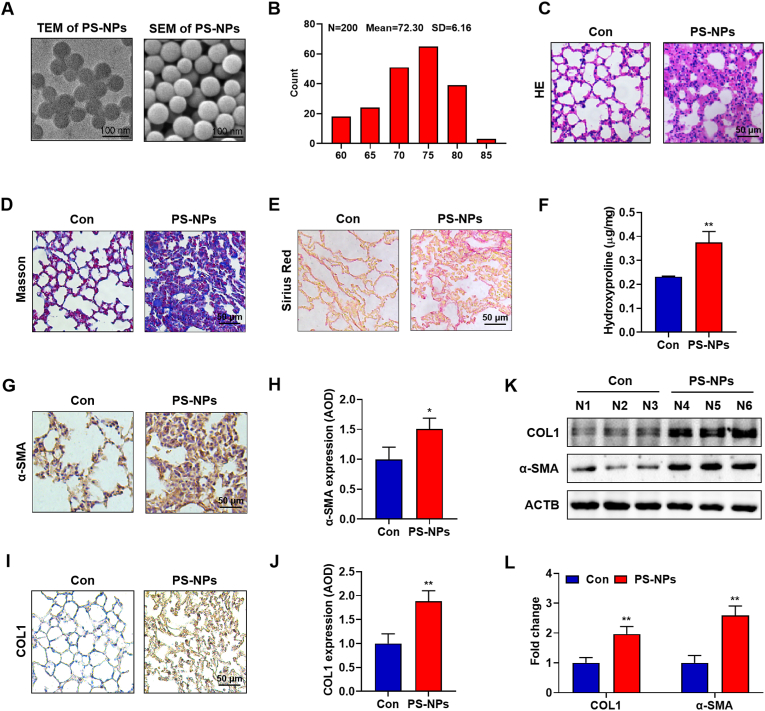
**PS-NPs Characterization and Induction of Pulmonary Fibrosis:**(A) TEM/SEM of spherical, monodisperse PS-NPs. (B) Size distribution (mean ± SD). (C) H&E staining showing alveolar collapse and septal thickening at day 28. (D–E) Masson’s trichrome (D) and Sirius Red (E) to confirm collagen accumulation. (F) Hydroxyproline assay quantifying total collagen. (G–J) α-SMA and COL1 IHC indicating myofibroblast activation. (K–L) Western blots of COL1 and α-SMA with densitometry. All in vivo experiments were performed with n = 8 mice per group. Statistical significance is denoted as *p < 0.05 and **p < 0.01. (For interpretation of the references to colour in this figure legend, the reader is referred to the Web version of this article.)

Representative TEM and SEM imaging demonstrated that the PS-NPs used in this study were spherical, monodisperse, and exhibited a mean diameter of 72.3 ± 6.16 nm (Fig. 1A and B), consistent with the nanoplastic fractions commonly identified in environmental samples [28]. After 28 days of oropharyngeal aspiration, H&E staining revealed pronounced alveolar shrinkage and inter-alveolar septal thickening in PS-NP-treated C57BL/6 mice (Fig. 1C). Collagen-specific Masson’s trichrome and Sirius Red staining revealed extensive fibrillar collagen deposition in PS-NPs–exposed lungs compared with controls (Fig. 1D and E). Consistently, biochemical quantification showed a significant increase in hydroxyproline content in the PS-NPs group (Fig. 1F). Immunohistochemistry further demonstrated marked upregulation of α-smooth muscle actin (α-SMA) (Fig. 1G and H) and collagen I (COL1) (Fig. 1I and J) in the lung interstitium, indicative of pronounced myofibroblast activation and extracellular matrix accumulation. These histological findings were corroborated by immunoblotting, which showed increased expression of both α-SMA and COL1 proteins in PS-NPs–treated lungs (Fig. 1K and L). To characterize the associated inflammatory response, we additionally examined cytokine expression in lung tissue (Supplementary Fig. 1). PS-NPs exposure significantly upregulated mRNA levels of IL-1β, IL-6, TNF-α, TGF-β and connective tissue growth factor (CTGF) (Fig. S1A–E). In line with these transcriptional changes, immunohistochemistry revealed increased protein expression of IL-6, TGF-β and TNF-α in PS-NPs–exposed lungs (Fig. S1F–H). Collectively, these data establish that repeated airway exposure to 80 nm PS-NPs is sufficient to trigger a robust fibrotic programme in murine lung, corroborating earlier reports that inhaled polystyrene microplastics promote collagen accumulation and pulmonary fibrosis [29].

### 3.2 PS-NPs trigger ferroptosis in lung epithelial cells driving pulmonary fibrosis

**Fig. 2:**
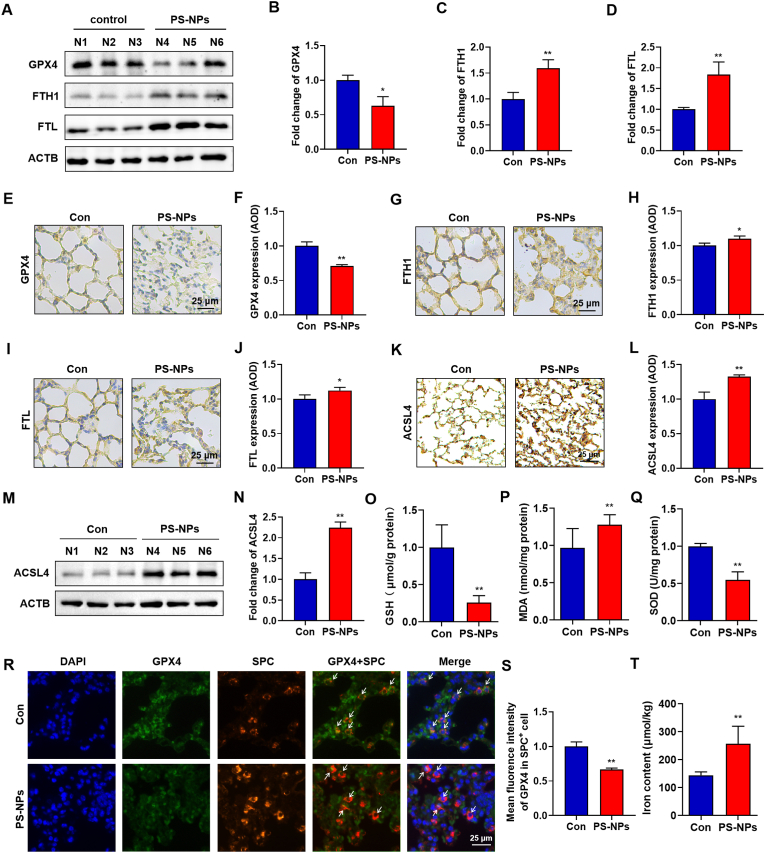
**Ferroptotic Alterations in PS-NPs–Exposed Mouse Lungs**: (A–D) Western blots and quantification of GPX4, FTH1, and FTL in lung homogenates. (E–J) IHC detection of GPX4, FTH1, and FTL in lung sections. (K–N) Western blots and IHC quantification of ACSL4 in lung. (O–Q) Biochemical measurements of GSH, SOD activity, and MDA levels. (R–S) Dual-label immunofluorescence for GPX4 in SPC^+^ AT2 cells (scale bar = 25 μm). (T) Total iron content assay of lung tissue. Statistical significance is denoted as *p < 0.05 and **p < 0.01.

Recent evidence identifies ferroptosis as a pivotal driver of idiopathic pulmonary fibrosis [30]. Consistently, fibrotic foci typically exhibit free-iron overload, elevated FTL and FTH1 expression, and suppressed GPX4 activity—hallmark indicators of ferroptosis [31]. Guided by these findings, we systematically examined ferroptosis related alterations in lungs from PS-NPs exposed mice. Western blotting of lung homogenates revealed a marked reduction of GPX4 protein, accompanied by robust upregulation of FTH1 and, in particular, FTL compared with controls (Fig. 2A–D). Consistently, IHC staining showed decreased GPX4 and increased FTH1 in the parenchyma, with FTL deposition rising even more prominently (Fig. 2E–J). To further substantiate a ferroptotic signature, we assessed ACSL4 and found that PS-NPs exposure significantly increased ACSL4 protein levels by western blot and IHC (Fig. 2K–N). Biochemical assays demonstrated pronounced depletion of GSH and SOD activity together with a sharp increase in MDA (Fig. 2O–Q), reinforcing the occurrence of oxidative stress–driven ferroptosis [32]. Dual-label immunofluorescence indicated a substantial loss of GPX4 signal within surfactant protein-C–positive type Ⅱ alveolar epithelial (AT2) cells (Fig. 2R and S), highlighting their particular vulnerability to nanoplastic-induced ferroptosis. Notably, AT2-cell ferroptosis has been implicated as a trigger for fibrotic remodeling [33]. Independent studies have likewise reported that PS-NPs damage airway and lung epithelial cells via oxidative-stress-ferroptosis pathways, lending further support to our observations [2]. Finally, total iron measurements confirmed a significant increase in lung iron content after PS-NPs exposure (Fig. 2T), fulfilling the iron overload prerequisite of ferroptosis. Collectively, PS-NPs exposure precipitates a canonical ferroptotic molecular landscape—GPX4 suppression, ferritin up-regulation, iron sequestration, and antioxidant exhaustion. The initiation of ferroptosis in AT2 cells may constitute a central event that drives subsequent collagen deposition and irreversible lung fibrosis.

### 3.3 Epithelial ferroptosis instigated by PS-NPs drives fibroblast activation through secreting pro-inflammatory and pro-fibrotic factors

Guided by our in vivo observation that AT2 cells are exquisitely sensitive to PS-NPs–induced ferroptosis, we turned to the BEAS-2B bronchial epithelial model. To assess nano-polystyrene uptake, the relative intracellular concentration of PS-NPs in BEAS-2B cells was quantified by measuring the phagocytosis of fluorescent polystyrene nanoplastics (80 nm) at 0, 6, 12 and 24 h using ImageJ (Fig. S2A and B). In parallel, potential cytotoxicity was evaluated by exposing BEAS-2B cells to increasing concentrations of PS-NPs (50, 100, 150 and 200 μg/mL), with cell viability assessed by CCK-8 assay. Based on this gradient test, 200 μg/mL was identified as a toxic dose (Fig. S2C). After 24 h exposure to 200 μg/ml PS-NPs, cells were subjected to both transcriptomic and proteomic profiling. RNA-seq identified 1853 differentially expressed genes (p < 0.05; Fig. S3A–E), among which KEGG analysis revealed significant enrichment of the ferroptosis pathway (Table S3). Intersection with known ferroptosis genes yielded 163 shared targets (Fig. S3C), and PPI network mapping showed that four of the 25 hub proteins were ferroptosis-related (Fig. S3D–F). Proteomics likewise uncovered 534 differentially abundant proteins (p < 0.05; Fig. S3G–H) with ferroptosis emerging as a top-ranked KEGG term (Table S4); PPI analysis revealed five ferroptosis nodes among 11 core interactors (Fig. S3I–K). Notably, FTL was robustly upregulated at protein levels, whereas FTH1 remained unchanged (Fig. S3L), directing us to focus subsequent mechanistic work on FTL.

To further define the role of FTL in this pathway, we performed siRNA mediated knockdown of FTL in BEAS-2B cells. Among the three FTL targeting siRNAs, siFTL3 achieved the highest knockdown efficiency and was selected for subsequent experiments (Fig. S4A and B). Under PS-NPs exposure, western blotting showed no obvious change in GPX4 protein levels between the siNC + PS and siFTL + PS groups (Fig. S4C and D). In contrast, C11-BODIPY staining revealed further increased lipid peroxidation in the siFTL + PS group, and FerroOrange fluorescence demonstrated greater accumulation of labile Fe^2+^ compared with siNC + PS (Fig. S4E and F). These findings indicate that FTL knockdown aggravates PS-NPs induced iron accumulation and lipid peroxidation even without additional suppression of GPX4, supporting FTL as a key protective molecule in this ferroptotic pathway.

Having established that FTL modulates the severity of ferroptotic injury, we next evaluated whether other cell death pathways contribute to PS-NPs induced cytotoxicity. We compared the effects of PS-NPs in the presence of inhibitors of ferroptosis, apoptosis or necroptosis in BEAS-2B cells. PS-NPs (200 μg/mL) markedly reduced cell viability, whereas co-treatment with Fer-1 provided the strongest rescue in the CCK-8 assay. By contrast, co-administration of the pan caspase inhibitor Z-VAD-FMK (20 μM) or the necroptosis inhibitor Necrostatin-1 (30 μM) produced little or no improvement in cell viability compared with PS-NPs alone (Fig. S4G). These data indicate that ferroptosis is the dominant mode of cell death under our experimental conditions, with apoptosis and necroptosis contributing only minimally to PS-NPs induced cytotoxicity in BEAS-2B cells.

**Fig. 3:**
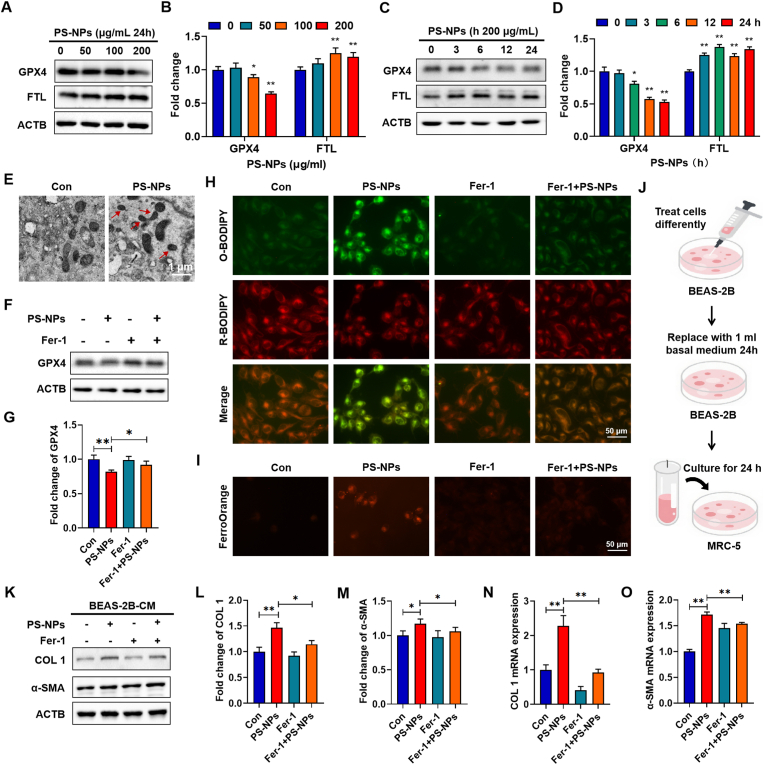
**PS-NPs-Induced Ferroptosis in BEAS-2B Cells and Paracrine Fibroblast Activation:** (A–B) Western blot of GPX4 and FTL in BEAS-2B cells after 24 h exposure to PS-NPs at 0, 50, 100, and 200 μg/ml (C–D) Time-course of GPX4 and FTL expression in cells treated with 200 μg/ml PS-NPs for 0, 3, 6, 12, and 24 h. (E) Transmission electron microscopy images of control and PS-NPs treated BEAS-2B cells showing characteristic mitochondrial shrinkage. (F–G) Restoration of GPX4 following co-treatment with Fer-1. (H) C11-BODIPY581/591 fluorescence showing Fer-1–mediated attenuation of lipid peroxidation. (I) FerroOrange staining demonstrating reduction of labile Fe^2+^ upon Fer-1 co-incubation. (J–O) COL1 and α-SMA mRNA (qPCR) and protein (western blot) levels in MRC-5 fibroblasts after 24 h treatment with conditioned medium from BEAS-2B cells exposed to PS-NPs ± Fer-1. Statistical significance is denoted as *p < 0.05 and **p < 0.01.

Consistent with these omics findings, western blotting demonstrated a dose-dependent decline in GPX4 paralleled by reciprocal FTL induction in BEAS-2B cells treated with PS-NPs (Fig. 3A and B), mirroring our mouse lung data. Time-course experiments (0–24 h) showed GPX4 suppression by 6 h and FTL overexpression as early as 3 h, persisting through 24 h (Fig. 3C and D), indicative of a rapid, sustained lipid-peroxidation response. Transmission electron microscopy further revealed ultrastructural features consistent with ferroptosis. In PS-NPs-treated BEAS-2B cells, mitochondria showed marked shrinkage, highlighted by red arrows (Fig. 3E). These mitochondrial changes indicate that PS-NPs induce a typical ferroptotic phenotype at the ultrastructural level. To confirm causality, co-treatment with the ferroptosis inhibitor Fer-1 fully restored GPX4 (Fig. 3F and G), abrogated lipid peroxidation as measured by C11-BODIPY581/591 (Fig. 3H), and reduced labile Fe^2+^ via FerroOrange staining (Fig. 3I).

Double immunofluorescence staining with SPC further indicates that alveolar epithelial cells can serve as an important cellular source of IL-6, TGF-β and TNF-α, which are key pro-fibrotic cytokines (Fig. S4H). To directly focus on the epithelial–fibroblast paracrine loop downstream of epithelial ferroptosis, MRC-5 cells were then incubated for 24 h with conditioned medium from BEAS-2B cells exposed to PS-NPs, with or without Fer-1 pretreatment (Fig. 3J). Conditioned medium from PS-NPs–treated epithelial cells elicited a marked upregulation of COL1 and α-SMA protein in MRC-5 fibroblasts (Fig. 3K–M), whereas Fer-1 pretreatment of BEAS-2B cells effectively prevented these protein increases. Importantly, parallel qRT-PCR analysis confirmed that Fer-1 likewise reversed the PS-NPs–induced elevations of COL1 and α-SMA transcripts (Fig. 3N and O). Moreover, KEGG analysis of our transcriptome data revealed significant enrichment of the ECM-receptor interaction pathway (Fig. S4I), providing a plausible link between epithelial ferroptosis and downstream fibroblast collagen synthesis.

Collectively, these integrated ‘omics and functional assays establish that PS-NPs provoke the canonical hallmarks of epithelial ferroptosis—GPX4 repression, FTL up-regulation, lipid peroxidation, and iron overload—and that ferroptotic epithelial cells, via secreted mediators, fuel fibroblast activation and ECM deposition; inhibition of ferroptosis effectively interrupts this paracrine profibrotic cascade.

### 3.4 YY1 governs transcriptional activation of FTL in PS-NP-exposed pulmonary epithelial cells

**Fig. 4:**
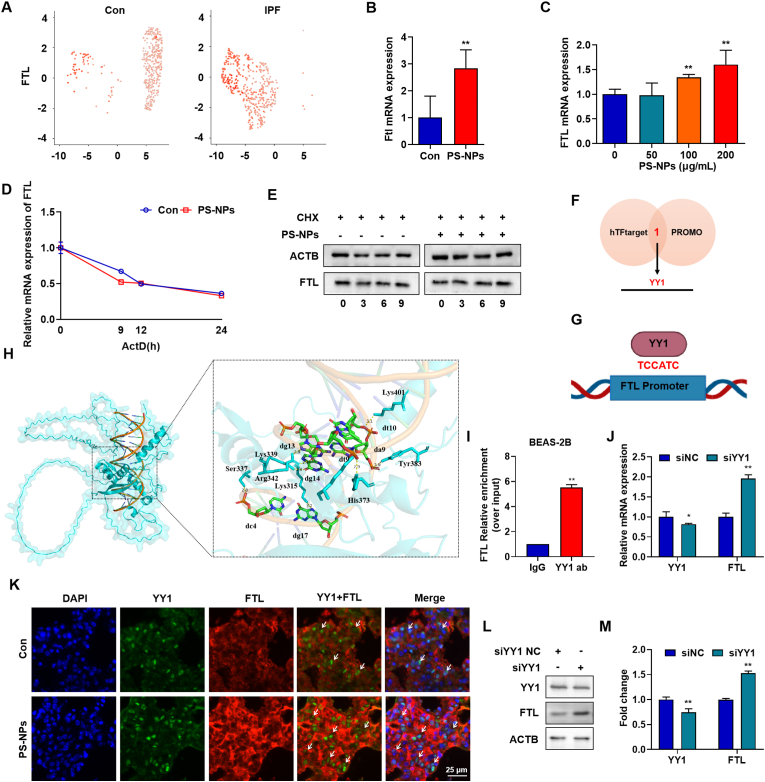
**Transcriptional Regulation of FTL by YY1 in PS-NPs-Exposed Lung:**(A) Single-cell RNA-seq analysis of FTL expression in alveolar epithelial cells from IPF patients versus healthy controls. (B) FTL mRNA levels in mouse lung tissue after 28 days of PS-NPs exposure (qRT-PCR). (C) Dose-dependent FTL mRNA induction in BEAS-2B cells treated with PS-NPs for 24 h. (D) Actinomycin D chase assay showing FTL mRNA decay kinetics following PS-NPs treatment. (E) Cycloheximide chase assay of FTL protein stability in PS-NPs–treated cells. (F–G) In silico prediction of upstream transcription factors using hTFtarget and PROMO, highlighting YY1 binding motifs in the FTL promoter (JASPAR). (H) Molecular docking simulation of YY1 interacting with the FTL promoter region. (I) ChIP assay validating YY1 binding to the FTL promoter. (J) qRT-PCR analysis of FTL mRNA levels in BEAS-2B cells after siRNA mediated YY1 knockdown. (K) Dual immunofluorescence showing YY1 and FTL co-expression in mouse lung sections (DAPI counterstain; scale bar = 25 μm). (L–M) Western blot and densitometric analysis of FTL protein levels in BEAS-2B cells after siRNA mediated YY1 knockdown. Statistical significance is denoted as *p < 0.05 and **p < 0.01.

To investigate the mechanisms underlying FTL elevation in PS-NPs-induced lung fibrosis, we first analyzed single-cell transcriptomes from the Human Lung Cell Atlas (HLCA) and found that FTL expression is significantly increased in alveolar epithelial cells of early-stage IPF patients (Fig. 4A; data from Sikkema et al., [34]. Consistent with this, PS-NPs exposure for 28 days raised FTL mRNA levels in both mouse lung tissue (Fig. 4B) and alveolar epithelial cells (Fig. 4C). To determine whether this up-regulation reflects changes in RNA or protein stability, we pretreated epithelial cells with actinomycin D and observed no alteration in FTL mRNA decay kinetics after PS-NPs (Fig. 4D), and similarly, cycloheximide chase assays revealed unchanged FTL protein stability (Fig. 4E). These findings imply that transcriptional activation could play a leading role in upregulating FTL. Using hTFtarget and PROMO, we identified YY1 as the sole transcription factor predicted by both platforms to bind the FTL promoter. JASPAR analysis further pinpointed a consensus YY1 motif (TCCATC) within the FTL promoter region (Fig. 4F and G). Molecular docking of AlphaFold3-modeled YY1 with the amplified FTL promoter fragment confirmed a stable interaction (Fig. 4H; Fig. S5A–C); mutation of TCCATC to TCGGTC abolished binding (ipTM = 0.62, pTM = 0.38; Fig. S5D and E). ChIP analysis showed significant enrichment of FTL promoter sequences in YY1 immunoprecipitates compared with IgG controls, confirming direct binding of YY1 to the FTL promoter in BEAS-2B cells (Fig. 4I). Functionally, siRNA mediated silencing of YY1 led to a marked increase in FTL mRNA levels (Fig. 4J) and FTL protein abundance (Fig. 4L and M). Double label immunofluorescence in mouse lung sections showed that regions with high YY1 signal had low FTL staining (Fig. 4K), consistent with a negative regulatory relationship between YY1 and FTL observed in vitro. To further support this link in human disease, analysis of GEO datasets (GSE110147, GSE213001, GSE231693) revealed a significant inverse correlation between YY1 and FTL expression in fibrotic lungs (Fig. S5F). To distinguish transcriptional regulation from potential effects on FTL protein stability, we performed a cycloheximide chase after YY1 silencing. YY1 knockdown transiently slowed FTL degradation at 6 h, but this difference disappeared by 9 h, indicating only a modest and short lived influence on FTL stability (Fig. S5G and H). Taken together, these results indicate that under PS-NPs exposure, YY1 primarily suppresses FTL expression through direct transcriptional control.

### 3.5 PS-NPs upregulate YY1 in pulmonary epithelia and murine lungs

**Fig. 5:**
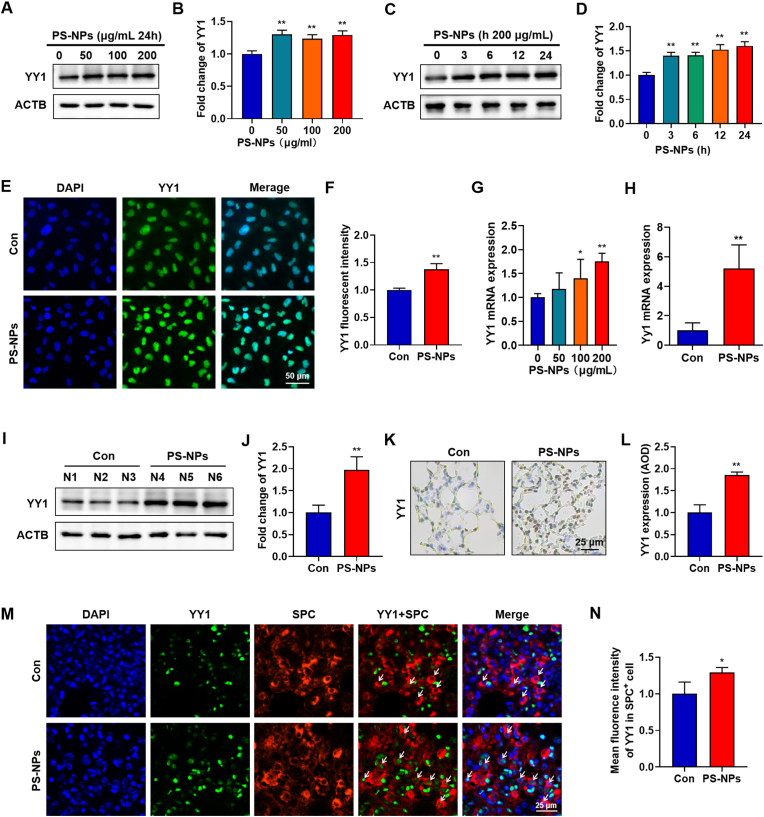
**PS-NPs Induce YY1 Up-regulation In Vitro and In Vivo:** (A–B) Western blot of YY1 in BEAS-2B cells after 24 h treatment with increasing PS-NPs concentrations. (C–D) Time-course analysis of YY1 protein in BEAS-2B cells exposed to 200 μg/ml PS-NPs. (E–F) Immunofluorescence showing nuclear localization and intensity of YY1 in BEAS-2B cells (scale bar = 50 μm). (G) qRT-PCR quantification of YY1 mRNA in BEAS-2B cells across PS-NPs dose gradient. (H) qRT-PCR measurement of YY1 mRNA in lung tissue. (I–J) Western blot and densitometric analysis of YY1 protein in whole-lung lysates. (K–L) IHC detection of YY1 in mouse lung sections following 28 days of PS-NPs exposure. (M − N) Dual-label immunofluorescence for YY1 in SPC^+^ AT2 cells (scale bar = 25 μm). (K) Statistical significance is denoted as *p < 0.05 and **p < 0.01.

Given that YY1 can directly bind the FTL promoter and regulate its transcriptional activity, we first profiled YY1 dynamics in BEAS-2B cells. A 24-h concentration-response experiment (0, 50, 100, 200 μg/ml) showed that YY1 protein was significantly elevated beginning at 50 μg/ml (Fig. 5A and B). In a separate time-course study, YY1 up-regulation was detectable as early as 3 h and continued to rise, reaching a peak at 24 h (Fig. 5C and D). Immunofluorescence confirmed that YY1 localized predominantly to the nucleus and that nuclear signal intensity increased markedly after PS-NPs exposure (Fig. 5E and F), consistent with its role as a transcription factor. RT-qPCR revealed a dose-dependent increase in YY1 mRNA, suggesting that PS-NPs enhance YY1 transcription rather than merely stabilizing the protein (Fig. 5G). In vivo, PS-NPs exposure for 28 days significantly increased YY1 mRNA levels in lung tissue, as shown by qRT-PCR (Fig. 5H). Consistently, western blotting of whole-lung lysates revealed elevated YY1 protein expression with corresponding densitometric analysis (Fig. 5I and J). IHC further confirmed enhanced YY1 staining in lung sections from PS-NPs–treated mice compared with controls (Fig. 5K and L). Moreover, dual-label immunofluorescence demonstrated intensified YY1 signal in SPC^+^ AT2 cells after PS-NPs exposure (Fig. 5M and N), indicating that alveolar epithelial cells are a major site of YY1 induction in vivo, in line with our in vitro findings. Notably, YY1 is also persistently up-regulated in classical bleomycin-induced models of pulmonary fibrosis and has been shown to promote fibroblast α-SMA expression and collagen deposition [35]. This parallels our observation of YY1 elevation and α-SMA induction following PS-NPs exposure, further underscoring YY1 as a pivotal driver across diverse profibrotic settings.

### 3.6 Knockdown of YY1 mitigates PS-NPs-mediated ferroptosis and fibrosis in vitro

**Fig. 6:**
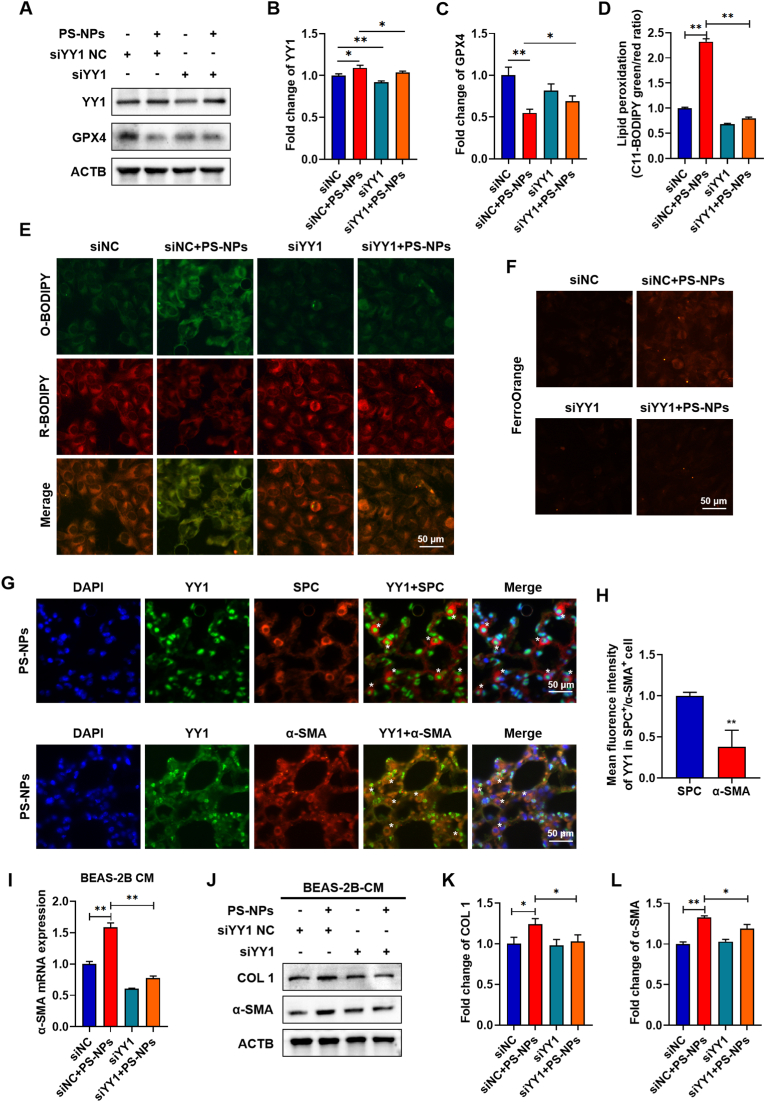
**YY1 Silencing Reverses PS-NPs–Induced Ferroptosis and Paracrine Fibrogenesis:** (A–C) Western blots showing restoration of GPX4 protein in BEAS-2B cells after YY1 knockdown and 24 h PS-NPs exposure. (D–E) C11-BODIPY581/591 fluorescence demonstrating reduced lipid peroxidation upon YY1 silencing. (F) FerroOrange staining indicating decreased labile Fe^2+^ accumulation after YY1 knockdown. (G–H) Double immunofluorescence staining of YY1 with the epithelial marker SPC and the myofibroblast marker α-SMA in lung sections from PS-NPs-treated mice. (I–L) Western blot and qRT-PCR analyses of COL1 and α-SMA in MRC-5 fibroblasts treated for 24 h with conditioned medium from BEAS-2B cells exposed to PS-NPs ± YY1 silencing. Statistical significance is denoted as *p < 0.05 and **p < 0.01.

To further define the role of YY1 along the nanoplastic ferroptosis fibrogenesis axis, we silenced YY1 in BEAS-2B cells with siRNA and then exposed the cells to PS-NPs for 24 h. Western blotting confirmed efficient YY1 knockdown and showed that loss of YY1 largely restored GPX4 protein expression under PS-NPs exposure (Fig. 6A–C). Consistently, C11-BODIPY581/591 staining revealed that YY1 silencing markedly reduced PS-NPs-induced lipid peroxidation, and FerroOrange staining showed a parallel decrease in labile Fe^2+^ (Fig. 6D–F), indicating that YY1 is required to sustain the GPX4 Fe^2+^ lipid peroxidation cascade. To identify the main cellular compartment in which YY1 is induced, we performed double immunofluorescence staining for YY1 with the epithelial marker SPC and the myofibroblast marker α-SMA. YY1 showed strong colocalization and marked signal changes with SPC, whereas its overlap with α-SMA was minimal (Fig. 6G and H), indicating that YY1 is predominantly upregulated in epithelial cells rather than fibroblasts in PS-NPs-induced pulmonary fibrosis. We next examined whether epithelial YY1 controls fibroblast activation through paracrine signaling. Conditioned medium from PS-NPs treated BEAS-2B cells induced a robust fibrogenic response in MRC-5 fibroblasts, with significant upregulation of COL1 and α-SMA mRNA and protein as measured by qRT-PCR and western blotting. In contrast, conditioned medium from YY1-silenced epithelial cells markedly blunted COL1 and α-SMA induction at both the transcript and protein level (Fig. 6I–L). This finding aligns with earlier work showing that YY1 up-regulation directly binds the COL1 promoter and drives myofibroblast differentiation [35]. Collectively, YY1 transcriptional status dictates the severity of PS-NPs–induced ferroptosis and amplifies epithelial–mesenchymal paracrine signaling to enhance downstream ECM synthesis; its knockdown not only lifts the ferroptotic block on GPX4 but also markedly dampens the coordinated up-regulation of α-SMA and COL1.

### 3.7 AAV-YY1 alleviates PS-NPs-induced pulmonary fibrosis by suppressing ferroptosis

**Fig. 7:**
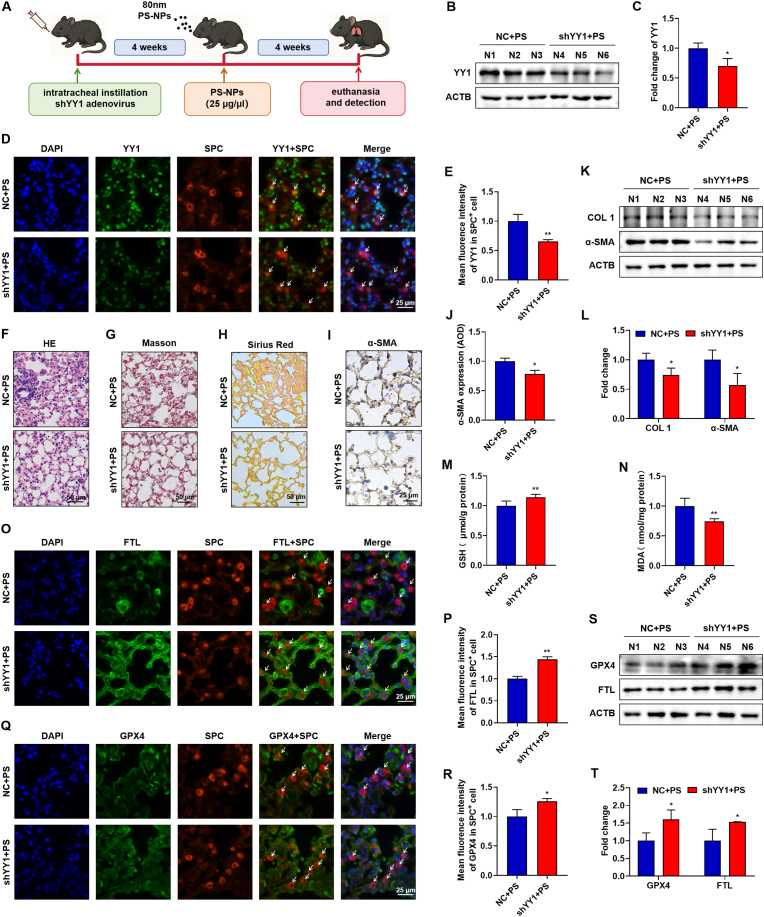
**YY1 knockdown in AT2 cells attenuates PS-NPs-induced pulmonary fibrosis in vivo:** (A) Schematic of intratracheal administration of spc-shYY1 adenovirus (≈6.28 × 10^10^ vg/mouse) for YY1 silencing in AT2 cells, followed by PS-NPs exposure. (B, C) Western blot confirming YY1 knockdown in lung tissue. (D, E) YY1/SP-C dual immunofluorescence validating YY1 reduction in AT2 cells. (F) H&E staining showing reduced interstitial thickening in the shYY1 + PS group. (G, H) Masson’s trichrome and Sirius Red staining demonstrate decreased collagen deposition. (I, J) α-SMA IHC indicates reduced myofibroblast activation. (K, L) Western blot analysis of COL1 and α-SMA protein levels. (M, N) GSH and MDA measurements showing restored antioxidant capacity and reduced lipid peroxidation. (O–R) Dual immunofluorescence showing YY1 knockdown restores GPX4 and further increases FTL in AT2 cells. (S, T) Western blot analysis of GPX4 and FTL expression in lung tissue. All in vivo experiments were performed with n = 8 mice per group. Statistical significance is denoted as *p < 0.05 and **p < 0.01. (For interpretation of the references to colour in this figure legend, the reader is referred to the Web version of this article.)

To determine whether epithelial YY1 is required for PS-NPs-induced lung injury, we generated AT2 cell specific YY1 knockdown by intratracheal delivery of spc-shYY1 adenovirus (≈6.28 × 10^10^ vg/mouse), followed by PS-NPs exposure (Fig. 7A). Western blotting of whole-lung lysates showed a clear reduction of YY1 protein in shYY1 + PS mice compared with NC + PS controls (Fig. 7B and C). Consistently, YY1/SP-C dual immunofluorescence and YY1 IHC confirmed efficient YY1 silencing in SP-C^+^ AT2 cells and in lung tissue (Fig. 7D and E; Fig. S6E and F). YY1 knockdown did not change body weight or wet-lung mass, but significantly reduced the lung-to-body-weight ratio and improved gross lung appearance (Fig. S6A–D), indicating attenuation of PS-NPs-induced remodeling. Histologically, H&E staining showed reduced interstitial thickening in the shYY1 + PS group (Fig. 7F), whereas Masson’s trichrome and Sirius Red staining demonstrated decreased collagen deposition (Fig. 7G and H). In line with this, α-SMA IHC revealed reduced myofibroblast activation (Fig. 7I and J), and western blotting confirmed lower COL1 and α-SMA protein levels (Fig. 7K and L), together with a significant decrease in total collagen content by hydroxyproline assay (Fig. S6G).

Biochemically, YY1 silencing restored GSH and reduced MDA in PS-NPs-exposed lungs (Fig. 7M and N), accompanied by recovery of SOD activity (Fig. S6L), indicating relief of oxidative stress. At the cellular level, dual immunofluorescence in SP-C^+^ AT2 cells showed that YY1 knockdown under PS-NPs exposure rescued GPX4 expression and further increased FTL staining (Fig. 7O–R). Consistently, western blot and IHC of whole-lung tissue demonstrated a robust rebound of GPX4 and FTL protein levels in the shYY1 + PS group (Fig. 7S and T; Fig. S6I–K). Total lung iron content was also significantly reduced (Fig. S6H), indicating mitigation of PS-NPs-induced iron overload. Together, these findings show that AT2 cell specific YY1 knockdown re-establishes antioxidant and iron homeostasis, limits GPX4-dependent ferroptosis, and markedly attenuates PS-NPs-induced pulmonary fibrosis, supporting YY1 as a potential therapeutic target in nanoplastic-related lung injury.

## 4 Discussion

The widespread presence of plastic particles, especially PS-NPs with smaller sizes, in the environment caused inevitable exposure of humans via air, water, and food, posing a growing concern for public health and safety [36,37]. Studies have shown that PS-NPs cause organ damage by distribution to multiple organs, such as the brain, heart, liver, and lung seem to be the primary target organs for the toxicological effects of PS-NPs [38,39]. Therefore, our study focused on the toxic effects of PS-NPs on the lung. Previous research has suggested that deposition PS-NPs respiratory tract can cause inflammatory responses, oxidative stress, apoptosis, and ER stress [40]. In the current study, we detected the potential hazards of PS-NPs on the lung and found that lung epithelial cell ferroptosis might be a significant cell death mechanism in the pathogenesis of pulmonary fibrosis triggered by PS-NPs.

Pulmonary fibrosis is increasingly recognized as a chronic consequence of persistent lung injury in which an epithelial to fibroblast paracrine loop plays a central role. Under fibrotic conditions, disruption of the basement membrane permits close crosstalk between epithelial cells and fibroblasts [41]. Injured or senescent ATII cells release TGF-β and a senescence-associated secretory phenotype rich in TNF-α, IL-6, IL-8 and CCL2, which drives fibroblast proliferation, myofibroblast differentiation and excessive matrix deposition [30,42,43]. In parallel, ferroptosis, an iron-dependent oxidative form of regulated cell death, has emerged as an immunogenic driver of lung inflammation, and pharmacological inhibition of ferroptosis attenuates experimental pulmonary fibrosis, underscoring the contribution of oxidative stress-driven epithelial death to fibrogenesis [30,44]. Within this framework, PS-NPs can be viewed as an upstream trigger that establishes a pro-inflammatory, pro-fibrotic microenvironment in the bronchoalveolar compartment. Prior studies show that inhaled polystyrene particles increase neutrophil influx, oxidative stress, inflammation and cytokine expression in the lung and bronchoalveolar lavage fluid [[45], [46], [47]]. Consistent with these reports, we found that PS-NPs exposure upregulated TGF-β, IL-6, TNF-α and IL-1β mRNA in lung tissue and increased TGF-β, IL-6 and TNF-α protein expression by IHC, while double immunofluorescence with SPC identified alveolar epithelial cells as a major source of these cytokines. Together, these data support a model in which PS-NPs induced epithelial damage, amplifying cytokine release from the alveolar epithelium, thereby reinforcing epithelial to fibroblast paracrine circuits that sustain fibroblast activation and progressive pulmonary fibrosis.

As a form of iron-dependent cell death driven by overwhelming lipid peroxidation, ferroptosis contributes to the pathogenesis of multiple lung disorders, including cancer, chronic obstructive pulmonary disease (COPD), and pulmonary fibrosis [48,49]. Ferroptosis is typically triggered through two distinct mechanisms: the endogenous pathway and the exogenous pathway [50]. The endogenous pathway is characterized by the accumulation of lipid peroxides, largely controlled by the ferroptosis regulator GPX4 [51,52]. The exogenous pathway, however, involves the activation of iron transporters and the suppression of ferritin, an iron storage protein complex composed of FTL and FTH1 [53]. FTL, as a core component of the ferritin complex, exerts a dual role in iron homeostasis [54]: under conditions of high intracellular iron, it sequesters iron within ferritin to limit oxidative damage [55], whereas under iron deficiency, it can facilitate iron release to support cellular metabolism [56]. In our model, PS-NPs exposure induced marked iron overload and lipid peroxidation in lung epithelial cells, accompanied by FTL upregulation; rather than representing a pro-ferroptotic signal, this FTL induction likely reflects a compensatory attempt to buffer excess iron. Consistent with the view that FTL upregulation usually represents a protective, iron-chelating response against ferroptosis [57], YY1 knockdown further enhanced FTL expression and significantly attenuated lipid ROS accumulation and cell death in PS-NPs–treated epithelial cells. Thus, in the context of PS-NPs–induced iron overload, FTL upregulation appears to be a compensatory but partially insufficient defense mechanism that can more effectively counteract ferroptosis when its induction is further amplified by relieving YY1-mediated repression.

Increasing evidence indicates that epithelial ferroptosis is not only a cell autonomous death program but also an active driver of lung fibrogenesis. In patients with pulmonary fibrosis and in bleomycin-induced models, iron deposition and ferroptotic death in alveolar type II cells promote fibroblast proliferation, myofibroblast differentiation and collagen deposition, whereas pharmacological inhibition of ferroptosis or iron chelation markedly attenuates fibrotic remodeling [58]. Mechanistically, ferroptotic cells undergo iron dependent lipid peroxidation and plasma membrane rupture, leading to the release of damage-associated molecular patterns such as HMGB1, as well as bioactive oxidized phospholipids and reactive aldehydes, which together amplify inflammatory and reparative responses in surrounding stromal cells [59]. In a nanoparticle induced lung injury model closely related to our system, HMGB1 released from injured bronchial epithelial cells after cobalt nanoparticle exposure was sufficient to activate lung fibroblasts via the HMGB1–RAGE–MAPK axis, resulting in increased expression of α-SMA and collagen I, whereas blockade of this pathway significantly reduced fibroblast activation [60]. Taken together, these findings support a model in which PS-NPs induced ferroptosis in alveolar epithelial cells drives fibroblast activation and pulmonary fibrosis through paracrine signals, including HMGB1, oxidized lipids and profibrotic cytokines.

We observed that exposure to PS-NPs triggers a rapid, coordinated up-regulation of FTL in both lung parenchymal cells and airway epithelium under iron-driven oxidative stress. As the iron-sequestering subunit of ferritin, FTL chelates ferrous iron (Fe^2+^) to mitigate lipid peroxidation [61]. Simultaneously, PS-NPs induce robust expression of the transcriptional repressor YY1 elevation that parallels its up-regulation during TGF-β-mediated fibrosis and epithelial-to-mesenchymal transition (EMT) [62]. Previous studies have shown that FTL is subject to finely tuned transcriptional control by multiple factors and is not governed by YY1 alone. In particular, HIF-1α binds directly to a hypoxia-responsive element (HRE) in the FTL promoter and markedly enhances its transcriptional activity; FTL expression positively correlates with HIF1A levels, indicating that HIF-1α is a strong positive driver of FTL upregulation [63]. Consistent with this, HIF1A is typically elevated in experimental models of pulmonary fibrosis [64,65], and our data similarly show increased HIF1A protein in PS-NPs–exposed lungs (Fig. S7A and B). In addition, the FTL promoter contains a canonical antioxidant response element (ARE) that is bound by NRF2. ChIP-seq and functional assays have demonstrated that NRF2 directly occupies the FTL promoter and induces its expression [66], and several studies have reported that NRF2 positively regulates FTL [67,68]. NRF2 itself is also commonly upregulated in pulmonary fibrosis models [69], in line with our observation that NRF2 protein is increased in PS-NPs–treated samples (Fig. S7C and D). Taken together, these findings suggest that in PS-NPs-induced pulmonary fibrosis, potent positive transcription factors such as HIF-1α and NRF2 are strongly activated and exert a sustained upward drive on FTL expression. Our data indicate that YY1, in this context, acts as a negative transcriptional regulator of FTL but constitutes only one inhibitory node within a broader regulatory network. Its repressive effect is partially overridden by the more dominant positive inputs from HIF-1α, NRF2 and possibly other activators. This integrated regulatory framework provides a coherent explanation for why YY1 and FTL are both increased after PS-NPs exposure and indicates that their concomitant upregulation is not paradoxical, but rather reflects the net outcome of opposing transcriptional forces acting on the FTL promoter.

Although YY1 is traditionally defined as a transcription factor, accumulating evidence indicates that it also contributes to pulmonary fibrosis through additional non-transcriptional mechanisms. In lung epithelial cells, YY1 not only regulates EMT-related gene expression downstream of TGF-β, but also modulates signaling crosstalk with NF-κB. For example, YY1 overexpression promotes nuclear translocation of NF-κB p65 during TGF-β-induced EMT, suggesting that YY1 functions as a signaling integrator at the interface of the TGF-β and NF-κB pathways rather than acting solely as a DNA-binding factor [62]. Moreover, in fibroblasts and idiopathic pulmonary fibrosis models, YY1 is regulated post-translationally by the E3 ubiquitin ligase NEDD4, which ubiquitinates YY1, alters its protein stability, and thereby modulates TAB1-dependent profibrotic proliferation and extracellular matrix production. Experimental manipulation of the NEDD4/YY1 axis significantly modifies fibrotic remodeling in vivo [70]. Taken together, these findings indicate that YY1 participates in the pathogenesis of pulmonary fibrosis through both direct transcriptional control of target genes and non-transcriptional mechanisms involving protein-protein interactions, signaling crosstalk, and ubiquitin proteasome-mediated regulation of YY1 abundance. Within this broader mechanistic framework, our data further support YY1 as a central node integrating multiple profibrotic signals in the injured lung.

In summary, our findings support a unified model in which PS-NPs act primarily on the lung epithelium to initiate a YY1-driven ferroptotic program that secondarily amplifies fibroblast activation and fibrosis through paracrine signaling. We show that repeated airway exposure to PS-NPs is sufficient to induce a fibrotic and inflammatory lung phenotype in vivo and to establish a canonical ferroptotic signature in AT2 and bronchial epithelial cells, characterized by GPX4 loss, ACSL4 induction, iron overload, lipid peroxidation, mitochondrial shrinkage and a compensatory but only partially effective increase in FTL. Integrated bioinformatic, biochemical and ChIP data identify YY1 as a direct transcriptional repressor of FTL, and PS-NPs exposure robustly upregulates YY1, particularly in SPC-positive epithelial cells. Genetic silencing of YY1 in epithelial cells derepresses FTL, restores GPX4, attenuates lipid ROS and Fe^2+^ accumulation, and markedly weakens the ability of epithelial conditioned medium to drive fibroblast COL1 and α-SMA induction, while pharmacologic inhibition of ferroptosis produces a similar loss of profibrotic paracrine activity. Crucially, AT2 cell specific YY1 knockdown in vivo re-establishes antioxidant and iron homeostasis, limits epithelial ferroptosis, and significantly reduces collagen deposition and myofibroblast expansion in PS-NPs-exposed lungs. Together, these results position epithelial YY1–FTL–GPX4 signaling as a central upstream axis that couples environmental nanoplastic exposure to ferroptosis dependent fibrotic remodeling and highlight epithelial YY1 and ferroptosis as attractive therapeutic targets for nanoplastic related lung fibrosis.

## CRediT authorship contribution statement

**Wenxia Bu:** Writing – original draft, Visualization, Project administration, Methodology, Investigation, Formal analysis, Data curation, Conceptualization. **Yueyuan Jin:** Visualization, Methodology, Investigation, Data curation. **Yifan Zhou:** Validation, Investigation. **Fengxu Wang:** Investigation, Formal analysis. **Dongnan Zheng:** Investigation. **Rongzhu Liu:** Investigation. **Xuehai Wang:** Investigation, Formal analysis. **Mengjiao Yu:** Investigation. **Shan Bao:** Investigation, Funding acquisition. **Rui Zhao:** Funding acquisition. **Jinlong Li:** Funding acquisition. **Xiaoyu Zhou:** Funding acquisition. **Jian Feng:** Funding acquisition. **Xinyuan Zhao:** Supervision, Resources, Methodology, Funding acquisition, Conceptualization. **Demin Cheng:** Writing – review & editing, Writing – original draft, Supervision, Resources, Project administration, Methodology, Funding acquisition, Conceptualization.

## Declaration of competing interest

The authors declare no competing interests.

## Data Availability

Data will be made available on request.
