# Supplementary material for: Polystyrene nanoplastics-induced lung epithelial cells ferroptosis promotes pulmonary fibrosis via YY1/FTL axis
Source: Mater Today Bio. 2025 Dec 24;36:102738. doi: 10.1016/j.mtbio.2025.102738 (PMC12813319; doi:10.1016/j.mtbio.2025.102738)
Supplement: Multimedia component 1 [file mmc1.docx]

**Polystyrene nanoplastics-induced lung epithelial cells ferroptosis promotes pulmonary fibrosis via YY1/FTL axis**

**Supplementary Fig. 1**

**
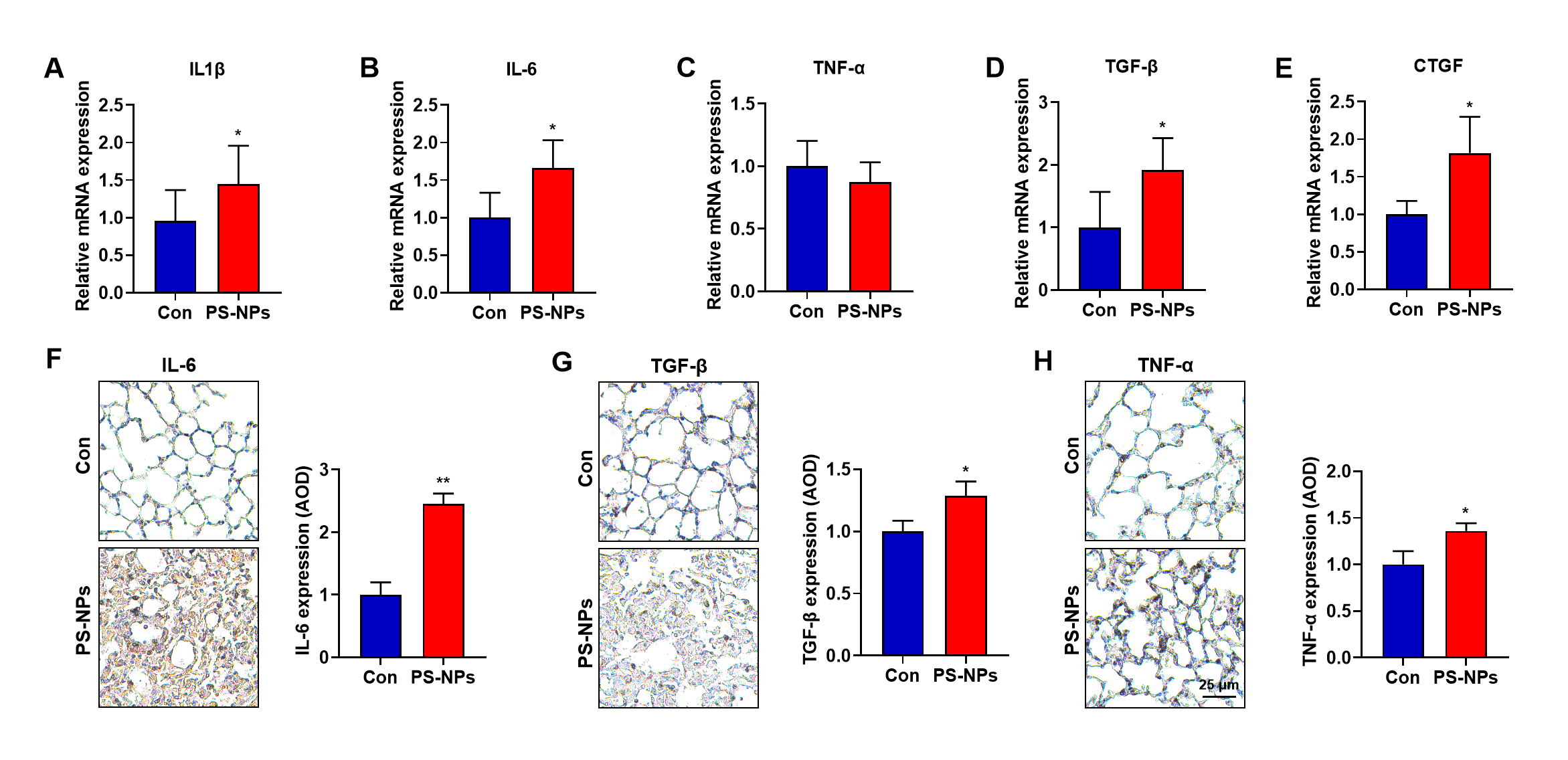
**

**Supplementary Fig. 1. PS-NPs exposure induces a pro-inflammatory, pro-fibrotic cytokine milieu in the lung.**

(A–E) qRT-PCR analysis showing that PS-NPs exposure upregulates IL-1β (A), IL-6 (B), TNF-α (C), TGF-β (D) and CTGF (E) mRNA levels in lung tissue. (F–H) Representative immunohistochemistry images demonstrating increased protein expression of IL-6 (F), TGF-β (G) and TNF-α (H) in PS-NPs–exposed lungs.

**Supplementary Fig. 2**

**
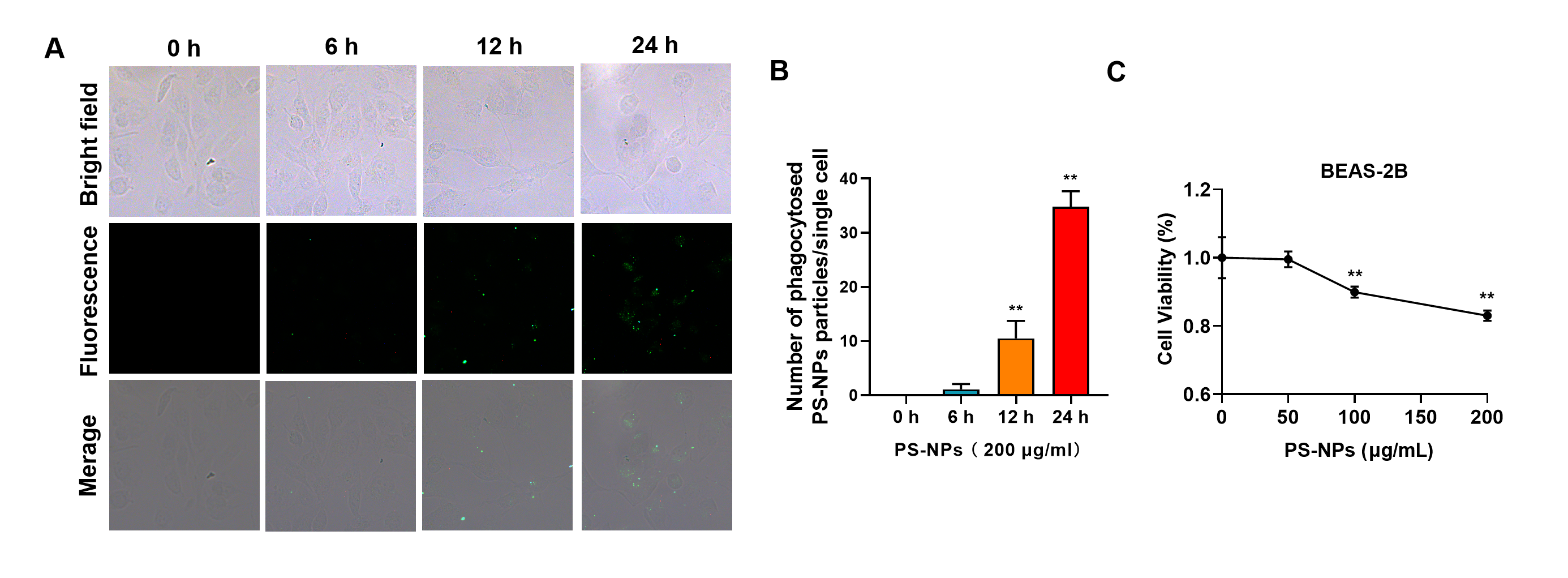
**

**Supplementary Fig. S2. Characterization of PS-NPs uptake and cell death modality in BEAS-2B cells.**

(A, B) Time-dependent uptake of fluorescent PS-NPs (80 nm) by BEAS-2B cells at 0, 6, 12 and 24 h. (C) CCK-8 assay showing cell viability after exposure to increasing PS-NPs concentrations (50, 100, 150 and 200 μg/mL).

**Supplementary Fig. 3**


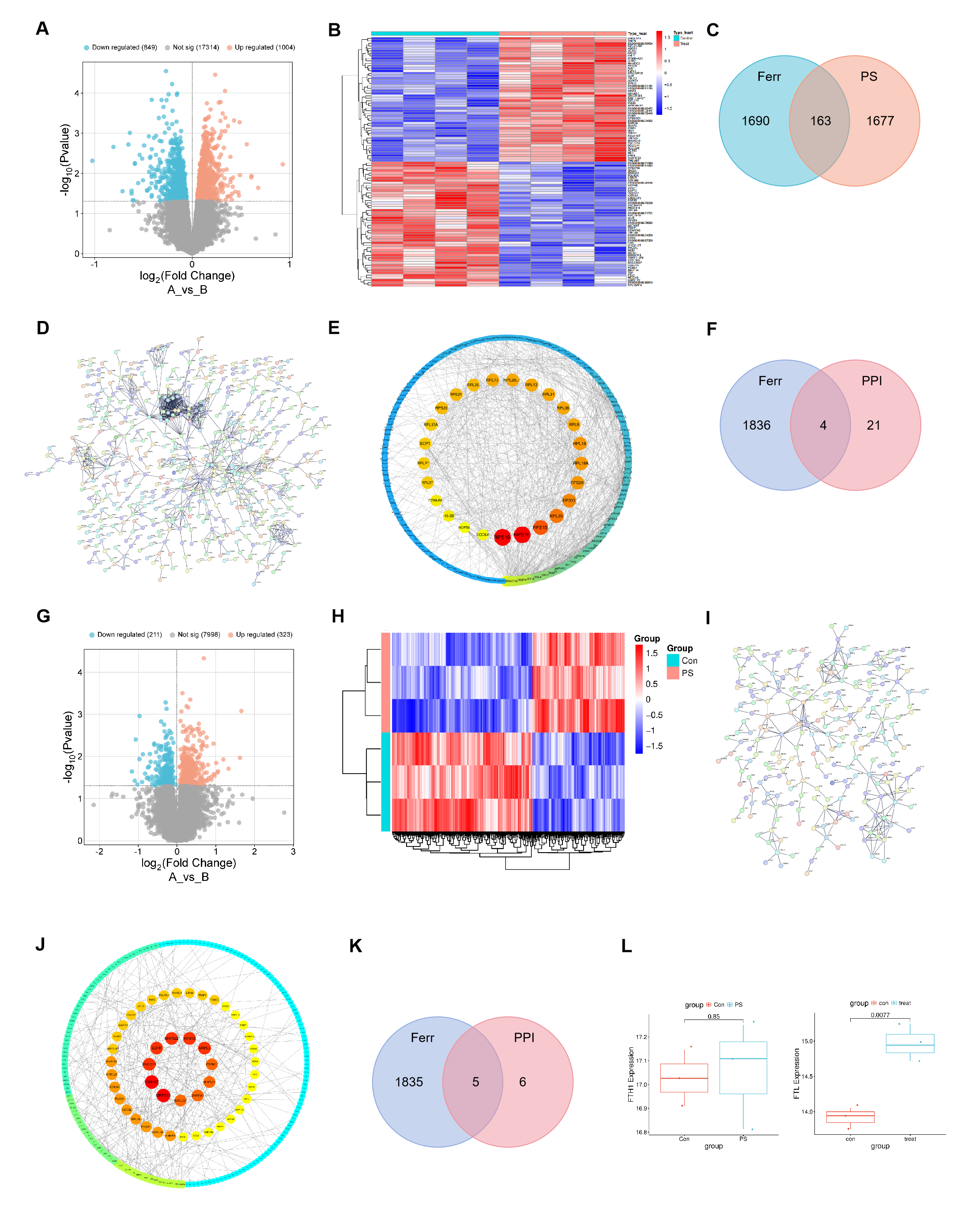


**Supplementary Fig. 3 Transcriptomic and Proteomic Profiling of PS‑NP‑Treated BEAS‑2B Cells**

(A) Volcano plot of differentially expressed genes from RNA‑seq. (B) Heatmap of top differentially expressed genes. (C) Venn diagram intersecting RNA‑seq DEGs with ferroptosis‑related genes. (D–E) PPI network of RNA‑seq DEGs (D) and zoom‑in view of ferroptosis‑related nodes (E). (F) Venn diagram of ferroptosis genes within the RNA‑seq PPI network. (G) Volcano plot of differentially expressed proteins from proteomics. (H) Heatmap of top differentially expressed proteins. (I–J) PPI network of proteomic DEPs (I) and magnified view of ferroptosis gene interactions (J). (K) Venn diagram of ferroptosis genes within the proteomic PPI network. (L) Proteomic quantification of FTL expression changes.

**Supplementary Fig. 4**

**
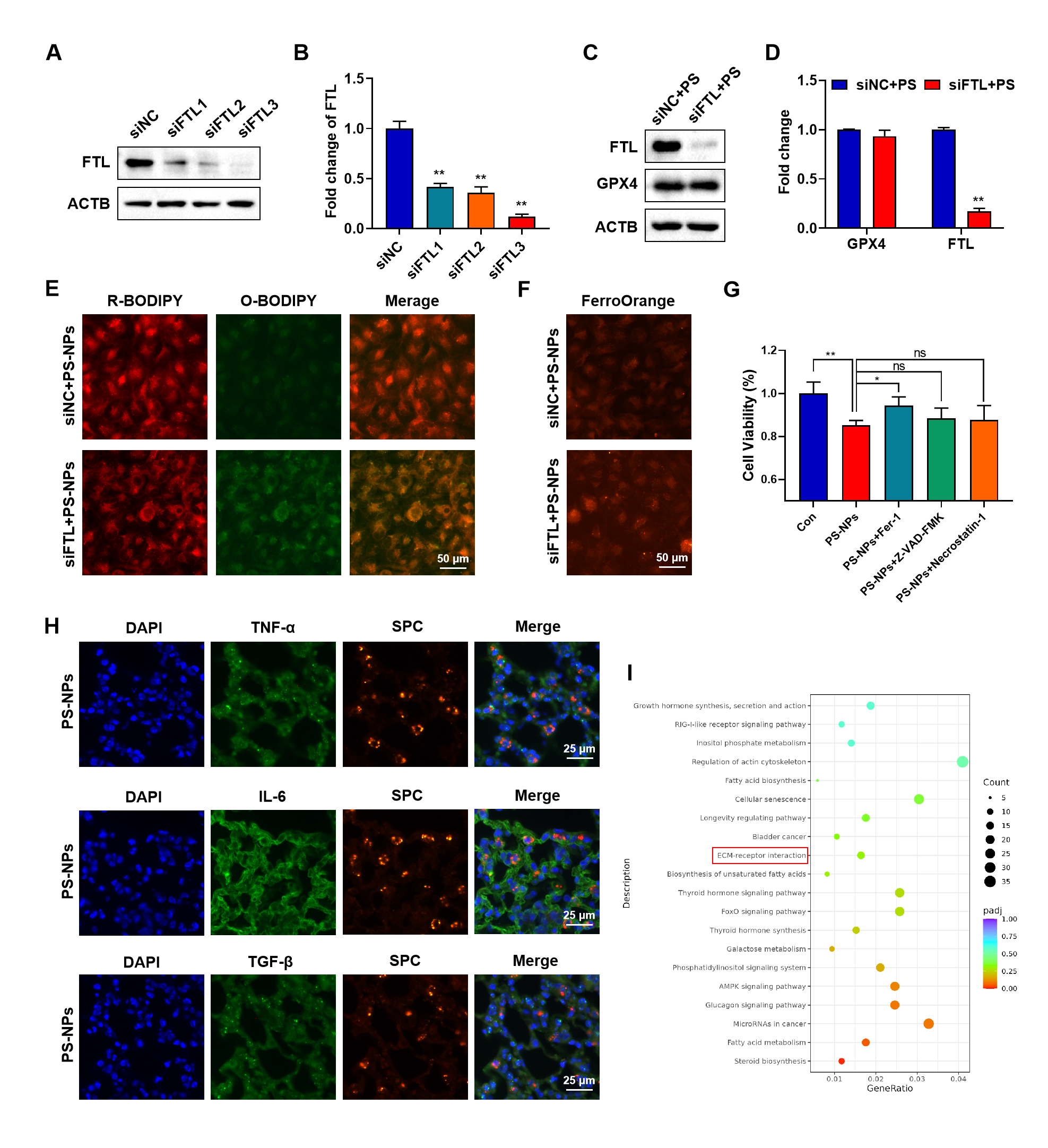
**

**Supplementary Fig. 4. Effects of FTL knockdown on ferroptosis in BEAS-2B**

(A, B) Western blot analysis of FTL showing siFTL3 as the most efficient siRNA. (C, D) Western blot of GPX4 protein in siNC+PS and siFTL+PS groups after PS-NPs exposure. (E) C11-BODIPY581/591 fluorescence showing increased lipid peroxidation in siFTL+PS cells. (F) FerroOrange staining showing enhanced labile Fe²⁺ accumulation in siFTL+PS cells. (G) Effects of Fer-1, Z-VAD-FMK and Necrostatin-1 on PS-NPs–induced loss of cell viability. (H) Double immunofluorescence staining for SPC and TGF-β, IL-6, and TNF-α showing their co-localization in alveolar epithelial cells. (I) KEGG pathway dot plot comparing PS‑NPs versus control.

**Supplementary Fig. 5**


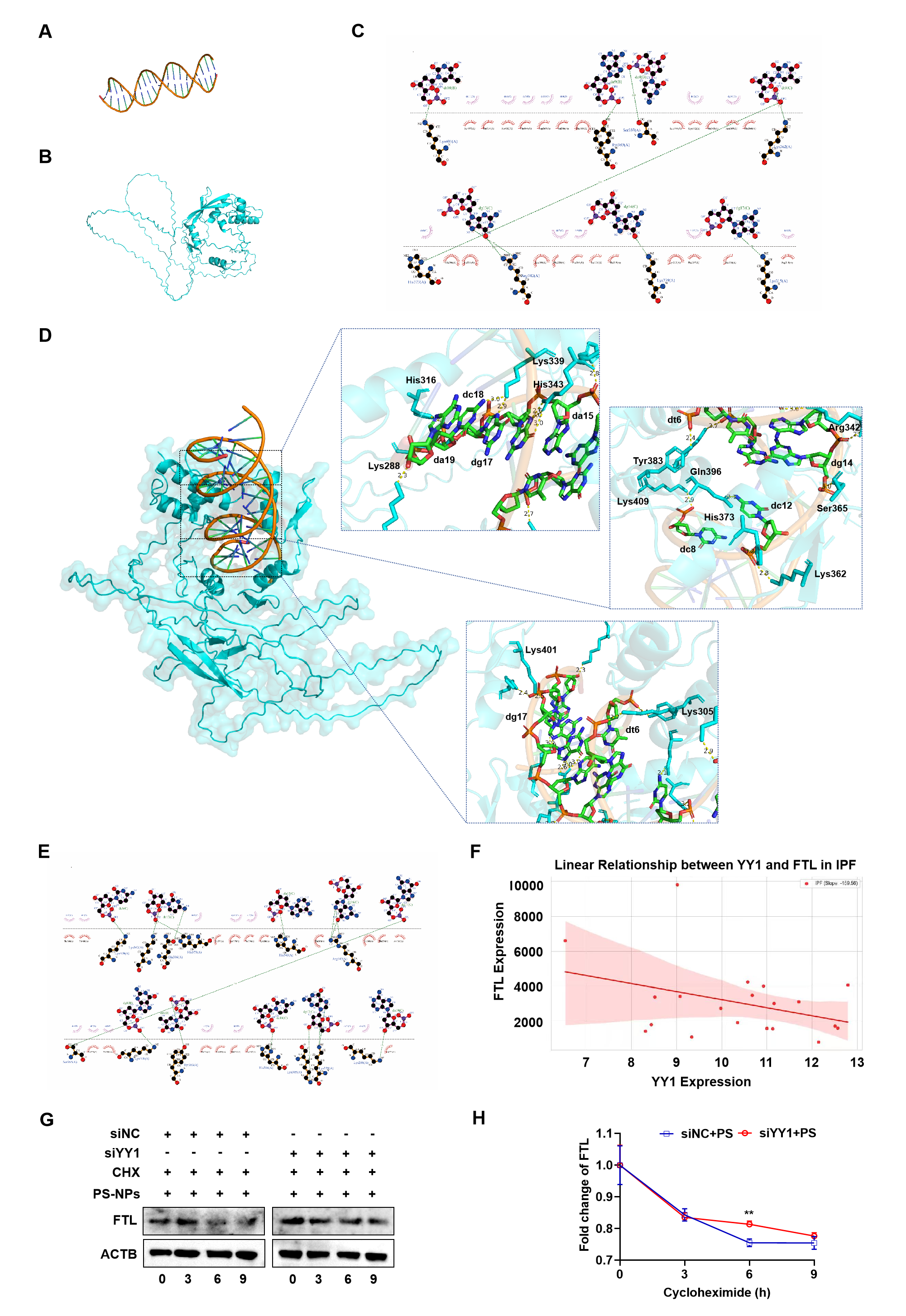


**Supplementary Fig. 5 In Silico Analysis of YY1–FTL Promoter Interactions**

(A) Diagram of the human FTL promoter fragment used for docking. (B) Ribbon model of the YY1 DNA‑binding domain. (C) Detailed interface between YY1 and the FTL promoter, with key contact residues highlighted (red) and hydrogen bonds shown as green dashed lines. (D–E) Local interaction maps of YY1 docked to two different mutated FTL promoter variants; yellow dashed lines denote hydrogen bonds, and annotated distances (Å) indicate bond lengths. (F) Correlation of YY1 and FTL expression in lung tissue from pulmonary fibrosis patients (GEO dataset). (G-H) Cycloheximide chase assay of FTL protein stability in siYY1+PS‑NPs treated cells.

**Supplementary Fig. 6**


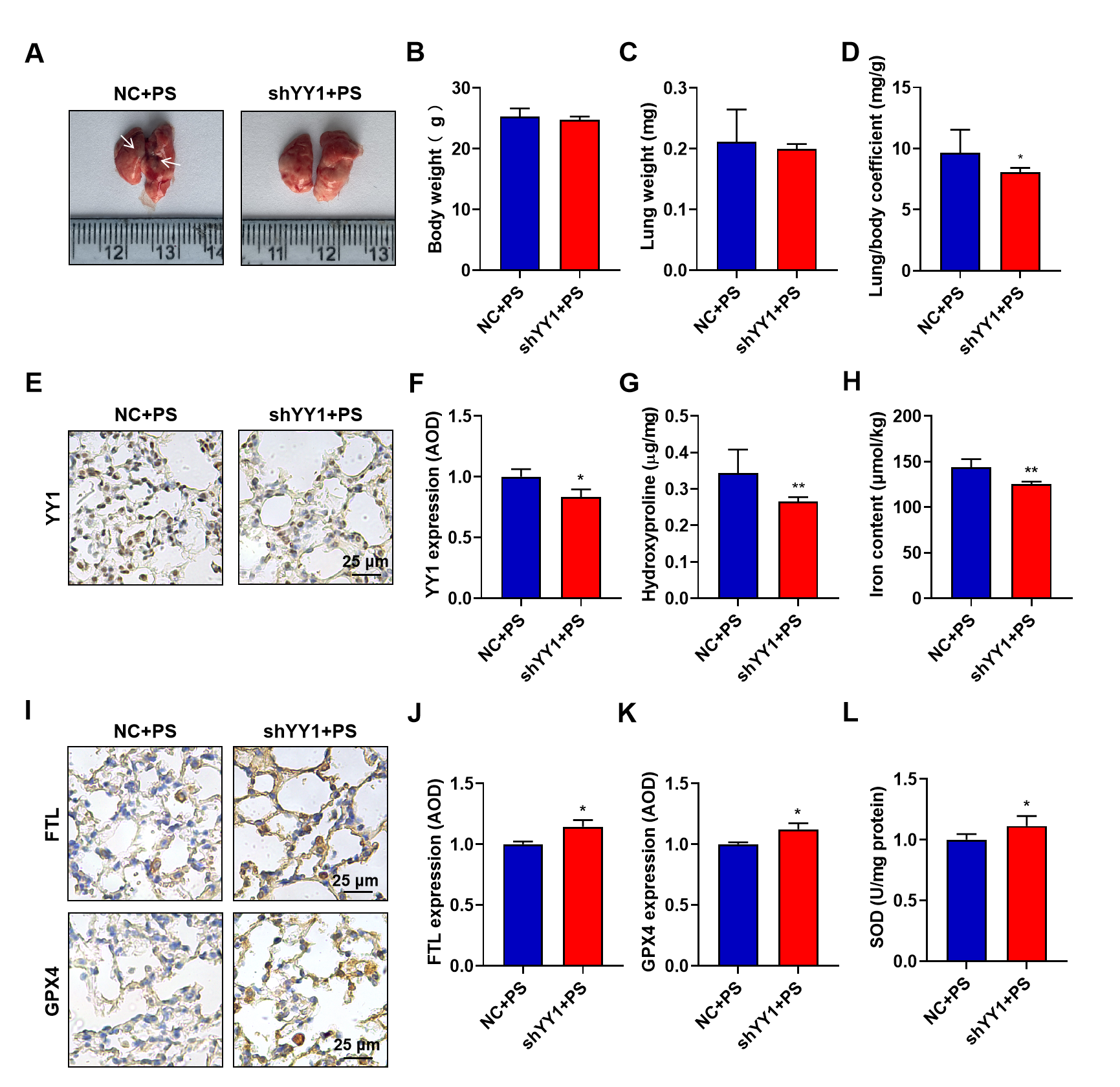


**Fig. S6. Additional characterization of YY1 knockdown in PS-NPs exposed mice:** (A-D) Gross lung appearance and lung-to-body-weight ratio after YY1 silencing and PS-NPs treatment. (E, F) IHC validation of YY1 knockdown in lung tissue. (G) Hydroxyproline assay quantifying total collagen content. (H) Total iron content in lung tissue. (I-K) IHC analysis of GPX4 and FTL expression. (L) SOD activity showing partial restoration after YY1 silencing.

**Supplementary Fig. 7**


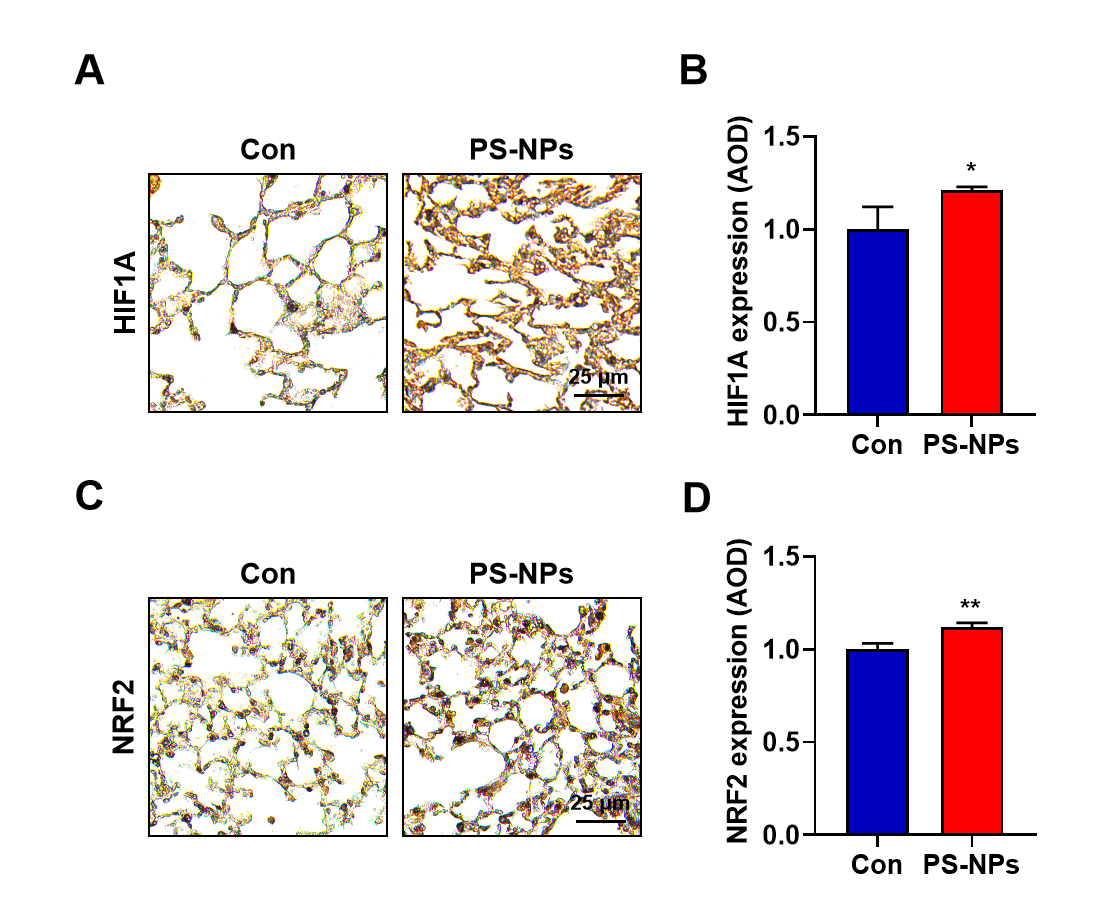


**Fig. S7. Expression of FTL related transcription factors in PS-NPs treated lungs：**

(A, B) IHC staining and quantification of HIF1A in lung sections from control and PS-NPs treated mice. (C, D) IHC staining and quantification of NRF2 in lung sections from control and PS-NPs treated mice.

**Supplementary Table 1. List of siRNA sequences.**

| Gene | sense(5’-3’) | antisense(5’-3’) |
| --- | --- | --- |
| FTL-Homo-255 | GCCUGGUCAAUUUGUACCUTT | AGGUACAAAUUGACCAGGCTT |
| FTL-Homo-426 | GCGCUCUCUUCCAGGACAUTT | AUGUCCUGGAAGAGAGCGCTT |
| FTL-Homo-685 | GGGCGAGUAUCUCUUCGAATT | UUCGAAGAGAUACUCGCCCTT |
| YY1-Homo | GUGGUUGAAGAACAGAUCAUUGGTT | CCAAUGAUCUGUUCUUCAACCACTT |

**Supplementary Table 2. List of primer sequences.**

| Gene | Forward primer | Reverse primer |
| --- | --- | --- |
| h- YY1 | ACGGCTTCGAGGATCAGATTC | TGACCAGCGTTTGTTCAATGT |
| m- YY1 | GAAAGCATCTGCACACCCAC | CTCCGGTATGGATTCGCACA |
| h- FTL | TACGAGCGTCTCCTGAAGATGC | GGTTCAGCTTTTTCTCCAGGGC |
| m- FTL | CCATCTGACCAACCTCCGC | CGCTCAAAGAGATACTCGCC |
| h- α-SMA | CCTGAAGAGCATCCCACCCT | ACCATCTCCAGAGTCCAGCACG |
| h- COL1 | TGACGAGACCAAGAACTGCC | GCACCATCATTTCCACGAGC |
| h- GAPDH | AATGGGCAGCCGTTAGGAAA | GCGCCCAATACGACCAAATC |
| m- GAPDH | TGGTCCAGGGTTTCTTACTC | GTTGTCTCCTGCGACTTCA |
| h-FTL-prom  -YY1 | CCCACGGCTCCAGAATAACT | AGGTTTGCGCTGCGAGATAA |

**Supplementary Table 3 RNA KEGG**

| **ID** | **Description** | **GeneRatio** | **BgRatio** | **pvalue** | **geneID** |
| --- | --- | --- | --- | --- | --- |
| hsa05171 | Coronavirus disease - COVID-19 | 25/278 | 238/9396 | 3.28803E-08 | RPL18/RPS19/TAB2/RPS20/RPL29/RPS29/RPL37A/RPL31/RPL8/NRP1/RPLP1/RPL35/RPL13/RPL26L1/RPL18A/JUN/RPL12/RPS15/RPL27/RPL37/RPS16/RPS28/RPL10A/RPLP2/RPL13A |
| hsa00100 | Steroid biosynthesis | 4/278 | 20/9396 | 0.002498212 | FDFT1/DHCR7/MSMO1/NSDHL |
| hsa01250 | Biosynthesis of nucleotide sugars | 5/278 | 37/9396 | 0.004388861 | GALK1/GALE/NANP/GNE/NAGK |
| hsa01212 | Fatty acid metabolism | 6/278 | 57/9396 | 0.006507904 | ACADVL/HACD2/ACACA/ACSL1/ELOVL6/FADS2 |
| hsa05219 | Bladder cancer | 5/278 | 41/9396 | 0.006855926 | MMP2/DAPK1/FGFR3/CCND1/HRAS |
| hsa05208 | Chemical carcinogenesis - reactive oxygen | 14/278 | 227/9396 | 0.00749107 | NDUFA11/ATP5MC3/MGST1/AHR/PPIF/JUN/HIF1A/COX5B/CAT/PTEN/NDUFV1/NDUFB2/AKR1C1/HRAS |
| hsa04962 | Vasopressin-regulated water reabsorption | 5/278 | 44/9396 | 0.009248302 | STX4/DCTN4/AQP3/CREB5/RAB11B |
| hsa04919 | Thyroid hormone | 9/278 | 122/9396 | 0.010109854 | ATP1B1/MED13L/TBC1D4/THRB/ITGAV/HIF1A/MED14/CCND1/HRAS |
| hsa04144 | Endocytosis | 14/278 | 252/9396 | 0.017554467 | VPS28/ARFGAP1/HLA-C/FGFR3/SPART/AP2B1/GRK6/PDCD6IP/ACTR3C/CBL/CYTH2/SH3GLB2/HRAS/RAB11B |
| hsa04218 | Cellular senescence | 10/278 | 157/9396 | 0.018080582 | GADD45A/HLA-C/EIF4EBP1/RRAS2/ITPR2/CCND1/PTEN/ETS1/ITPR1/HRA |
| hsa05230 | Central carbon metabolism in cancer | 6/278 | 71/9396 | 0.018258219 | FGFR3/HIF1A/LDHA/PTEN/GLS/HRAS |
| hsa04210 | Apoptosis | 9/278 | 137/9396 | 0.020312005 | GADD45A/BCL2L11/JUN/FADD/ITPR2/LMNB2/ITPR1/HRAS/ENDOG |
| hsa04820 | Cytoskeleton in muscle cells | 13/278 | 232/9396 | 0.020351096 | ATP1B1/FHL1/TMOD3/SNTA1/ITGA1/MYH15/SNTB2/ITGAV/COL11A2/SDC4/LMNB2/FHL2/COL11A1 |
| hsa05216 | Thyroid cancer | 4/278 | 37/9396 | 0.023011698 | GADD45A/CCND1/NCOA4/HRAS |
| hsa04621 | NOD-like receptor signaling | 11/278 | 189/9396 | 0.02466929 | TAB2/IFI16/TRIP6/JUN/FADD/CARD6/ITPR2/NLRP1/ITPR1/TP53BP1/PKN2 |
| hsa00520 | Amino sugar and nucleotide sugar metabolism | 4/278 | 38/9396 | 0.025138477 | GALK1/GALE/GNE/NAGK |
| hsa04140 | Autophagy - animal | 10/278 | 169/9396 | 0.028428901 | DAPK1/ATG16L2/HIF1A/RRAS2/PIK3R4/TP53INP2/PTEN/ITPR1/ATG4B/HRAS |
| hsa00900 | Terpenoid backbone biosynthesis | 3/278 | 23/9396 | 0.029296655 | HMGCR/HMGCS1/PCYOX1 |
| hsa05213 | Endometrial cancer | 5/278 | 59/9396 | 0.029873404 | GADD45A/CCND1/PTEN/APC/HRAS |
| hsa05166 | Human T-cell leukemia virus 1 infection | 12/278 | 224/9396 | 0.034119875 | HLA-C/NRP1/JUN/MAP3K1/CANX/  FOSL1/RANBP1/CCND1/PTEN/ETS1/CREB5/HRAS |
| hsa04216 | Ferroptosis | 4/278 | 42/9396 | 0.03480 | STEAP3/ACSL1/GSS/NCOA4 |
| hsa05205 | Proteoglycans in cancer | 11/278 | 204/9396 | 0.039842048 | MMP2/ITGAV/LUM/SDC4/HIF1A/CBL/  RRAS2/ITPR2/CCND1/ITPR1/HRAS |
| hsa04068 | FoxO signaling pathway | 8/278 | 133/9396 | 0.043565006 | GADD45A/FBXO32/BCL2L11/CCND1/  CAT/PTEN/SGK2/HRAS |
| hsa05210 | Colorectal cancer | 6/278 | 87/9396 | 0.043757015 | GADD45A/BCL2L11/JUN/CCND1/APC/HRAS |
| hsa01040 | Biosynthesis of unsaturated fatty acids | 3/278 | 27/9396 | 0.044402608 | HACD2/ELOVL6/FADS2 |
| hsa04912 | GnRH signaling pathway | 6/278 | 93/9396 | 0.057211894 | MMP2/JUN/MAP3K1/ITPR2/ITPR1/HRAS |
| hsa05418 | Fluid shear stress and atheroscleros | 8/278 | 142/9396 | 0.05971602 | MMP2/MGST1/ITGAV/ACVR1/SDC4/  JUN/BMPR2/ARHGEF2 |
| hsa00270 | Cysteine and methionine metabolism | 4/278 | 52/9396 | 0.067165305 | CBS/MAT2A/LDHA/GSS |
| hsa04152 | AMPK signaling | 7/278 | 122/9396 | 0.069795575 | HMGCR/EIF4EBP1/ACACA/CCND1/  PFKFB4/CREB5/RAB11B |
| hsa01230 | Biosynthesis of amino acids | 5/278 | 75/9396 | 0.070840046 | CBS/PYCR3/MAT2A/ASL/ASNS |
| hsa04918 | Thyroid hormone synthesis | 5/278 | 75/9396 | 0.070840046 | ATP1B1/CANX/ITPR2/ITPR1/CREB5 |
| hsa04810 | Regulation of actin cytoskeleton | 11/278 | 232/9396 | 0.08340746 | ITGA1/FGFR3/ITGAV/PIKFYVE/IQGAP3/ACTR3C/RRAS2/MYLK/APC/SSH2/HRAS |
| hsa03015 | mRNA surveillance pathway | 6/278 | 103/9396 | 0.084596866 | RNMT/FUS/RNPS1/DAZAP1/PABPC1L/NXT1 |
| hsa01521 | EGFR tyrosine kinase inhibitor resistance | 5/278 | 80/9396 | 0.087885023 | FGFR3/EIF4EBP1/BCL2L11/PTEN/HRAS |
| hsa00512 | Mucin type O-glycan biosynthesis | 3/278 | 36/9396 | 0.089523136 | B4GALT5/GCNT1/GALNT10 |
| hsa04137 | Mitophagy - animal | 6/278 | 105/9396 | 0.090813475 | TOMM7/JUN/HIF1A/RRAS2/PINK1/HRAS |
| hsa04625 | C-type lectin receptor signaling pathway | 6/278 | 105/9396 | 0.090813475 | JUN/RRAS2/MALT1/ITPR2/ITPR1/HRAS |
| hsa04974 | Protein digestion and absorption | 6/278 | 105/9396 | 0.090813475 | COL12A1/ATP1B1/SLC7A8/COL11A2/  SLC38A2/COL11A1 |
| hsa00250 | Alanine, aspartate and glutamate metabolism | 3/278 | 37/9396 | 0.095391792 | ASL/ASNS/GLS |
| hsa00480 | Glutathione metabolism | 4/278 | 59/9396 | 0.096571648 | MGST1/ODC1/OPLAH/GSS |
| hsa04110 | Cell cycle | 8/278 | 158/9396 | 0.096725358 | GADD45A/PKMYT1/SGO1/AURKB/CCND1/PDS5B/MCM5/ATRX |
| hsa04922 | Glucagon signaling pathway | 6/278 | 107/9396 | 0.097271862 | ACACA/LDHA/ITPR2/ITPR1/SIK2/CREB5 |
| hsa00061 | Fatty acid biosynthesis | 2/278 | 18/9396 | 0.097833856 | ACACA/ACSL1 |
| hsa00541 | Biosynthesis of various nucleotide sugars | 2/278 | 20/9396 | 0.116972677 | NANP/GNE |
| hsa04915 | Estrogen signaling pathway | 7/278 | 139/9396 | 0.118031365 | MMP2/GABBR1/JUN/ITPR2/ITPR1/  CREB5/HRAS |
| hsa04022 | cGMP-PKG signaling pathway | 8/278 | 166/9396 | 0.119217339 | ATP1B1/KCNMA1/ATP2B4/PPIF/ITPR2/  MYLK/ITPR1/CREB5 |
| hsa04371 | Apelin signaling pathway | 7/278 | 140/9396 | 0.121304519 | RRAS2/ITPR2/PIK3R4/CCND1/MYLK/  ITPR1/HRAS |
| hsa04727 | GABAergic synapse | 5/278 | 89/9396 | 0.123384635 | GABBR1/SLC6A12/SLC38A2/GLS/  GABRE |
| hsa05022 | Pathways of neurodegeneration - multiple diseases | 19/278 | 483/9396 | 0.124860652 | NDUFA11/DCTN3/ATP5MC3/FUS/PPIF/  DCTN4/FADD/ITPR2/PIK3R4/COX5B/  CAT/TRAP1/ITPR1/NDUFV1/PINK1/APC/NDUFB2/CSF1/HRAS |
| hsa04929 | GnRH secretion | 4/278 | 65/9396 | 0.125784726 | GABBR1/ITPR2/ITPR1/HRAS |

**Supplementary Table 4 Protein KEGG**

| **ID** | **Description** | **GeneRatio** | **BgRatio** | **pvalue** | **geneID** |
| --- | --- | --- | --- | --- | --- |
| hsa05160 | Hepatitis C | 17/269 | 159/9396 | 2.8148E-06 | APAF1/STAT3/PIK3R1/YWHAQ/YWHAH/CD81/IFIT1/RB1/CASP9/EIF3E/MAVS/YWHAZ/PPP2CB/SOS1/IKBKG/TRAF6/EGFR |
| hsa01200 | Carbon metabolism | 14/269 | 117/9396 | 5.82277E-06 | ACAT1/DLAT/PKLR/IDH3A/SUCLG1/MMUT/ADPGK/PSPH/GOT1/ESD/SDHB/PHGDH/OGDHL/IDH2 |
| hsa00020 | Citrate cycle (TCA cycle) | 7/269 | 30/9396 | 1.68709E-05 | DLAT/IDH3A/SUCLG1/SDHB/OGDHL/IDH2/ACLY |
| hsa04114 | Oocyte meiosis | 14/269 | 139/9396 | 4.22653E-05 | YWHAQ/YWHAH/ITPR2/FBXW11/PPP2R5B/CAMK2D/ANAPC7/BUB1/ANAPC16/ITPR1/YWHAZ/PPP2CB/MAPK12/ADCY9 |
| hsa04931 | Insulin resistance | 11/269 | 109/9396 | 0.000276009 | PTPA/SREBF1/PYGL/STAT3/PTPN11/PIK3R1/TBC1D4/INSR/PRKCE/PPP1R3D/TRIB3 |
| hsa05169 | Epstein-Barr virus infection | 15/269 | 204/9396 | 0.000755192 | APAF1/STAT3/PIK3R1/SNW1/USP7/PSMC4/RB1/CCNA2/ICAM1/CASP9/PSMC3/MAVS/MAPK12/IKBKG/TRAF6 |
| hsa05161 | Hepatitis B | 13/269 | 163/9396 | 0.000796136 | APAF1/STAT3/PIK3R1/YWHAQ/RB1/CCNA2/CASP9/MAVS/YWHAZ/SOS1/MAPK12/IKBKG/TRAF6 |
| hsa05131 | Shigellosis | 17/269 | 253/9396 | 0.000949229 | FOXO3/PIK3R1/CASP4/ACTG1/ITPR2/MAP1LC3A/FBXW11/PRKCE/ATG5/PPID/WASF1/ITPR1/PLCG1/MAPK12/IKBKG/TRAF6/EGFR |
| hsa05223 | Non-small cell lung cancer | 8/269 | 73/9396 | 0.001091517 | STAT3/FOXO3/PIK3R1/RB1/CASP9/PLCG1/SOS1/EGFR |
| hsa01524 | Platinum drug resistance | 8/269 | 75/9396 | 0.001304254 | APAF1/PIK3R1/MGST1/CASP9/TOP2A/MLH1/MSH6/GSTP1 |
| hsa05417 | Lipid and atherosclerosis | 15/269 | 216/9396 | 0.001350696 | APAF1/APOA1/STAT3/PIK3R1/HSPD1/ICAM1/CASP6/CASP9/CAMK2D/ITPR1/PLCG1/MAPK12/IKBKG/TRAF6/TNFRSF10B |
| hsa05213 | Endometrial cancer | 7/269 | 59/9396 | 0.001393122 | FOXO3/PIK3R1/CASP9/MLH1/SOS1/AXIN1/EGFR |
| hsa04110 | Cell cycle | 12/269 | 158/9396 | 0.001910088 | YWHAQ/YWHAH/RB1/CCNA2/PPP2R5B/ANAPC7/BUB1/ANAPC16/YWHAZ/PPP2CB/AURKB/MAD2L1BP |
| hsa04750 | Inflammatory mediator regulation of TRP channels | 9/269 | 99/9396 | 0.002063412 | PIK3R1/ITPR2/PRKCE/CAMK2D/ITPR1/  PLCG1/IL1RAP/MAPK12/ADCY9 |
| hsa00620 | Pyruvate metabolism | 6/269 | 47/9396 | 0.002086226 | ACAT1/DLAT/PKLR/HAGH/ALDH9A1/  LDHA |
| hsa01210 | 2-Oxocarboxylic acid metabolism | 5/269 | 33/9396 | 0.002279498 | DLAT/IDH3A/GOT1/OGDHL/IDH2 |
| hsa05210 | Colorectal cancer | 8/269 | 87/9396 | 0.003366198 | PIK3R1/CASP9/RALA/MLH1/MSH6/  SOS1/AXIN1/EGFR |
| hsa03430 | Mismatch repair | 4/269 | 23/9396 | 0.003785565 | LIG1/MLH1/MSH6/RPA1 |
| hsa05235 | PD-L1 expression and PD-1 checkpoint pathway in cancer | 8/269 | 90/9396 | 0.004149701 | STAT3/PTPN11/PIK3R1/PLCG1/MAPK12/IKBKG/TRAF6/EGFR |
| hsa01230 | Biosynthesis of amino acids | 7/269 | 75/9396 | 0.005505048 | PKLR/IDH3A/PSPH/GOT1/PHGDH/IDH2/MAT2A |
| hsa05205 | Proteoglycans in cancer | 13/269 | 204/9396 | 0.005770864 | FZD2/RDX/STAT3/PTPN11/PIK3R1/ACTG1/ITPR2/CAMK2D/ITPR1/PLCG1/SOS1/MAPK12/EGFR |
| hsa05203 | Viral carcinogenesis | 13/269 | 205/9396 | 0.006008241 | STAT3/PIK3R1/SNW1/YWHAQ/YWHAH/GTF2E1/USP7/RB1/CCNA2/YWHAZ/MRPS18B/DNAJA3/IKBKG |
| hsa05212 | Pancreatic cancer | 7/269 | 77/9396 | 0.006356286 | STAT3/PIK3R1/RB1/CASP9/RALA/IKBKG/EGFR |
| hsa04722 | Neurotrophin signaling pathway | 9/269 | 120/9396 | 0.007425099 | FOXO3/PTPN11/PIK3R1/CAMK2D/PLCG1/SOS1/MAPK12/TRAF6/ARHGDIA |
| hsa04621 | NOD-like receptor signaling pathway | 12/269 | 189/9396 | 0.008081404 | CASP4/DNM1L/ITPR2/MAP1LC3A/ATG5/ITPR1/MAVS/PKN2/MAPK12/IKBKG/TRAF6/DHX33 |
| hsa05170 | Human immunodeficiency virus 1 infection | 13/269 | 213/9396 | 0.008200657 | GNG5/PIK3R1/ITPR2/FBXW11/SAMHD1/CASP9/ITPR1/PLCG1/TRIM5/CUL5/MAPK12/IKBKG/TRAF6 |
| hsa04213 | Longevity regulating pathway - multiple species | 6/269 | 62/9396 | 0.008367162 | FOXO3/PIK3R1/CLPB/INSR/ATG5/ADCY9 |
| hsa05225 | Hepatocellular carcinoma | 11/269 | 170/9396 | 0.009618621 | FZD2/PIK3R1/ACTG1/RB1/LRP5/MGST1/PLCG1/SOS1/AXIN1/GSTP1/EGFR |
| hsa04072 | Phospholipase D signaling pathway | 10/269 | 149/9396 | 0.010436519 | PTPN11/PIK3R1/INSR/PLPP1/F2/RALA/PLCG1/SOS1/EGFR/ADCY9 |
| hsa05167 | Kaposi sarcoma associated herpesvirus infection | 12/269 | 196/9396 | 0.010630261 | GNG5/STAT3/PIK3R1/ITPR2/RB1/MAP1LC3A/ICAM1/CASP9/ITPR1/PLCG1/MAPK12/IKBKG |
| hsa04980 | Cobalamin transport and metabolism | 3/269 | 18/9396 | 0.01376976 | CD320/MMUT/ZNF143 |
| hsa05110 | Vibrio cholerae infection | 5/269 | 51/9396 | 0.014879647 | ACTG1/ATP6V0A1/PLCG1/TJP2/ADCY9 |
| hsa05120 | Epithelial cell signaling in Helicobacter pylori infection | 6/269 | 71/9396 | 0.015768885 | PTPN11/ATP6V0A1/PLCG1/MAPK12/  IKBKG/EGFR |
| hsa04210 | Apoptosis | 9/269 | 137/9396 | 0.01676852 | APAF1/PIK3R1/ACTG1/ITPR2/CASP6/  CASP9/ITPR1/IKBKG/TNFRSF10B |
| hsa04520 | Adherens junction | 7/269 | 93/9396 | 0.017062887 | ACTG1/PTPRJ/INSR/NECTIN2/WASF1/  CTNND1/EGFR |
| hsa04912 | GnRH signaling pathway | 7/269 | 93/9396 | 0.017062887 | ITPR2/CAMK2D/ITPR1/SOS1/MAPK12/  EGFR/ADCY9 |
| hsa05222 | Small cell lung cancer | 7/269 | 93/9396 | 0.017062887 | APAF1/PIK3R1/RB1/CASP9/LAMB2/  IKBKG/TRAF6 |
| hsa04918 | Thyroid hormone synthesis | 6/269 | 75/9396 | 0.020184333 | ATP1B1/ITPR2/CANX/ITPR1/ALB/  ADCY9 |
| hsa04120 | Ubiquitin mediated proteolysis | 9/269 | 142/9396 | 0.020690623 | PRPF19/FBXW11/HUWE1/SAE1/ANAPC7/ANAPC16/UBA2/CUL5/TRAF6 |
| hsa04971 | Gastric acid secretion | 6/269 | 76/9396 | 0.021407056 | ATP1B1/ACTG1/ITPR2/CAMK2D/ITPR1/ADCY9 |
| hsa05214 | Glioma | 6/269 | 76/9396 | 0.021407056 | PIK3R1/RB1/CAMK2D/PLCG1/SOS1/  EGFR |
| hsa01212 | Fatty acid metabolism | 5/269 | 57/9396 | 0.023116324 | ACAT1/PPT2/ACSL4/ACAA1/MCAT |
| hsa04152 | AMPK signaling pathway | 8/269 | 122/9396 | 0.023704535 | SREBF1/FOXO3/PIK3R1/INSR/CCNA2/  PPP2R5B/CAB39/PPP2CB |
| hsa04660 | T cell receptor signaling pathway | 8/269 | 122/9396 | 0.023704535 | PTPN11/PIK3R1/PPP2R5B/PLCG1/  PPP2CB/SOS1/MAPK12/IKBKG |
| hsa04935 | Growth hormone synthesis, secretion and action | 8/269 | 122/9396 | 0.023704535 | STAT3/PIK3R1/ITPR2/ITPR1/PLCG1/  SOS1/MAPK12/ADCY9 |
| hsa04933 | AGE-RAGE signaling pathway in diabetic complication | 7/269 | 101/9396 | 0.025602031 | STAT3/PIK3R1/F3/ICAM1/PRKCE/  PLCG1/MAPK12 |
| hsa01521 | EGFR tyrosine kinase inhibitor resistance | 6/269 | 80/9396 | 0.026797175 | STAT3/FOXO3/PIK3R1/PLCG1/SOS1/  EGFR |
| hsa05163 | Human cytomegalovirus infection | 12/269 | 226/9396 | 0.029113669 | GNG5/STAT3/PIK3R1/ITPR2/RB1/CASP9/ITPR1/SOS1/MAPK12/IKBKG/EGFR/  ADCY9 |
| hsa05208 | Chemical carcinogenesis - reactive oxygen species | 12/269 | 227/9396 | 0.029984605 | FOXO3/PTPN11/PIK3R1/PTPRJ/MGST1/  SDHB/NDUFA5/SOS1/MAPK12/ATP5PD/  IKBKG/EGFR |
| hsa04137 | Mitophagy - animal | 7/269 | 105/9396 | 0.030809617 | FOXO3/MAP1LC3A/TOMM7/FIS1/  HUWE1/MTX1/TFE3 |
